# Comparison of Cardiovascular Events Among Users of Different Classes of Antihypertension Medications: A Systematic Review and Network Meta-analysis

**DOI:** 10.1001/jamanetworkopen.2019.21618

**Published:** 2020-02-21

**Authors:** Jingkai Wei, Karla I. Galaviz, Alysse J. Kowalski, Matthew J. Magee, J. Sonya Haw, K. M. Venkat Narayan, Mohammed K. Ali

**Affiliations:** 1 Department of Epidemiology, Milken Institute School of Public Health, George Washington University, Washington, DC; 2 Emory Global Diabetes Research Center, Hubert Department of Global Health, Rollins School of Public Health, Emory University, Atlanta, Georgia; 3 Nutrition and Health Sciences Doctoral Program, Laney Graduate School, Emory University, Atlanta, Georgia; 4 Division of Endocrinology, Metabolism and Lipids, Department of Medicine, School of Medicine, Emory University, Atlanta, Georgia; 5 Department of Family and Preventive Medicine, School of Medicine, Emory University, Atlanta, Georgia

## Abstract

**Importance:**

Antihypertension medications have been associated with prevention of cardiovascular events, although less is known about the comparative effectiveness of different medication classes.

**Objective:**

To compare contemporary aggregated first-in-trial cardiovascular events among patients with hypertension and no substantial comorbidities.

**Data Sources:**

The PubMed, Embase, and Cochrane Library databases were systematically searched for articles published between January 1, 1990, and October 24, 2017.

**Study Selection:**

Randomized clinical trials that tested commonly used antihypertension medications (angiotensin-converting enzyme inhibitors, dihydropyridine calcium channel blockers, nondihydropyridine calcium channel blockers, β-blockers, angiotensin receptor blockers, and diuretics) and that reported selected cardiovascular outcomes for at least 6 months of follow-up.

**Data Extraction and Synthesis:**

The analysis was conducted from October 2017 to December 2019. Two reviewers extracted the number of cardiovascular events at the end of treatment for all study groups. For each outcome, a frequentist network meta-analysis was used to compare risk reductions between medication classes (random-effects models weighted by the inverse variance). The dose-response association between a 10–mm Hg reduction of systolic blood pressure and a 5–mm Hg reduction of diastolic blood pressure and the risk of first-in-trial cardiovascular events was estimated.

**Main Outcomes and Measures:**

First-in-trial cardiovascular events, including cardiovascular death, myocardial infarction, stroke, and revascularization.

**Results:**

In this systematic review and network meta-analysis, data were pooled from 46 eligible clinical trials (248 887 total participants with a mean [SD] age of 65.6 [5.8] years; 52.8% men). In the network meta-analysis, compared with placebo, angiotensin-converting enzyme inhibitors, dihydropyridine calcium channel blockers, and thiazide diuretics were reported to be similarly effective in reducing overall cardiovascular events (25%), cardiovascular death (20%), and stroke (35%); angiotensin-converting enzyme inhibitors were reported to be the most effective in reducing the risk of myocardial infarction (28%); and diuretics were reported to be the most effective in reducing revascularization (33%). In the metaregression analyses, each 10–mm Hg reduction in systolic blood pressure and 5-mm Hg reduction in diastolic blood pressure was significantly associated with a lower risk of cardiovascular death, stroke, and overall cardiovascular events.

**Conclusions and Relevance:**

In this network meta-analysis of clinical trials of patients with hypertension and no substantial comorbidities, different classes of antihypertension medications were associated with similar benefits in reducing cardiovascular events. Future studies should compare the effectiveness of combinations of antihypertension medications in reducing cardiovascular events.

## Key Points

**Question:**

Which classes of antihypertension medications are reported to be associated with reductions in first-in-trial cardiovascular events?

**Findings:**

In this systematic review and network meta-analysis of 46 randomized clinical trials that performed direct comparisons of individual antihypertension medication classes among 248 887 patients with hypertension and no substantial comorbidities, angiotensin-converting enzyme inhibitors, dihydropyridine calcium channel blockers, and diuretics were reported to be similarly effective in reducing cardiovascular death, stroke, and overall cardiovascular events. Angiotensin-converting enzyme inhibitors and diuretics were reported to be the most effective in reducing myocardial infarction and revascularization, respectively.

**Meaning:**

The reported effects of different antihypertension medication classes were largely similar, with only nuanced differences.

## Teaser

This systematic review and network meta-analysis examines randomized clinical trials of antihypertension medication classes from 1990 to 2017 to assess which medication classes are reported to be the most effective in reducing first-in-trial cardiovascular events among patients with hypertension.

## Introduction

Hypertension is the most prevalent risk factor for mortality and disability-adjusted life-years^1^ worldwide. Cardiovascular disease remains the leading cause of death globally, accounting for 17.7 million deaths in 2015, which represented 31% of all deaths worldwide.^2^ Hypertension is an important factor in cardiovascular disease.^3^ In 2010, it was estimated that one-third of the world’s adult population had hypertension.^4^ The introduction of the American College of Cardiology and the American Heart Association hypertension guidelines in 2017 resulted in higher estimates of the number of people with hypertension.^5,6^ Knowledge of optimal first-line antihypertension medications for the prevention of cardiovascular events and mortality will be important for clinical decision-making. Moreover, the identification of treatments that are most effective for controlling hypertension and subsequent cardiovascular events and mortality and that have the least harmful effects is imperative to guide clinicians and decrease cardiovascular disease burdens worldwide.

Previous meta-analyses have examined the efficacy of antihypertension treatments in reducing cardiovascular events.^7,8^ These meta-analyses have used pairwise comparisons of only 2 classes of antihypertension medications; however, pairwise meta-analysis does not enable comparison of multiple classes of medications. Only 1 network meta-analysis^9^ has compared the effectiveness of different classes of antihypertension medications in preventing cardiovascular events, but this meta-analysis was published more than 15 years ago and included medications, such as α-blockers, that are less frequently used in contemporary health care.

To provide an updated perspective on the comparative efficacy of antihypertension medications, we conducted a network meta-analysis to compare the reported effects of different classes of antihypertension medications that are currently used to reduce the risk of individual cardiovascular events (cardiovascular death, myocardial infarction, stroke, and revascularization) and to examine the medications’ reported effectiveness in reducing the overall risk of any cardiovascular event. Findings from this study will be relevant for the contemporary clinical management of hypertension, especially in light of the new American College of Cardiology and American Heart Association hypertension guidelines.^10^

## Methods

We conducted a network meta-analysis of randomized clinical trials that tested antihypertension medications. This study followed the Preferred Reporting Items for Systematic Reviews and Meta-analyses (PRISMA) guidelines for systematic reviews and meta-analyses.^11^

### Data Sources and Study Selection

We systematically searched the PubMed, Embase, and Cochrane Library databases for articles published between January 1, 1990, and October 24, 2017. The search terms used included hypertension, hypertension and agents, and antihypertensive agents (a detailed list of search terms is available in the eMethods in the Supplement. There were no language restrictions.

We included studies meeting the following criteria: (1) randomized clinical trials; (2) published during or after 1990; (3) included nonpregnant adults 18 years or older without chronic kidney disease, diabetic nephropathy, or organ transplants (which are risk factors for secondary hypertension) and without myocardial infarction and/or stroke within the previous 6 months; (4) evaluated antihypertension medications, including angiotensin-converting enzyme (ACE) inhibitors, dihydropyridine calcium channel blockers (DH CCBs), nondihydropyridine calcium channel blockers (non–DH CCBs), β-blockers, angiotensin receptor blockers (ARBs), and diuretics compared with control groups receiving placebo, standard treatment, or health education; (5) reported incidence of cardiovascular disease events (cardiovascular death, myocardial infarction, stroke, and/or coronary revascularization [either percutaneous coronary intervention or coronary artery bypass grafting]); and (6) reported data from at least 6 months of follow-up. In addition, we excluded studies that reported myocardial infarction and/or stroke within the previous 3 months; studies that focused on patients with chronic kidney disease, diabetic nephropathy, or organ transplants (which are risk factors for secondary hypertension); and studies that were not focused on patients with hypertension. To minimize concerns about the heterogeneity of outcome ascertainment across studies, we only included studies in which cardiovascular events were adjudicated by physicians using similar criteria and assessing patients’ medical records.

Study selection followed 3 steps. First, 2 of us (J.W. and A.J.K.) independently screened the titles of studies. Second, the same 2 reviewers screened and selected abstracts, and disagreements were resolved by a third reviewer (M.K.A.). Third, the 2 reviewers (J.W. and A.J.K.) examined the full text of articles for confirmation of inclusion. Disagreements were resolved by consensus or, when necessary, by the third reviewer (M.K.A.).

### Data Extraction and Quality Assessment

Two of us (J.W. and A.J.K.) independently extracted data from studies that met inclusion criteria using a standardized extraction form. The data extracted included sample size, participant characteristics (age and sex), study country, follow-up duration, types of antihypertension medications and comparator groups, and number of first-in-trial cases for each outcome.

Cardiovascular death was defined as death related to cardiovascular disease or death that could be calculated using all-cause mortality minus noncardiovascular-related death. We used nonfatal myocardial infarction and stroke if they were indicated or could be calculated, and we used total (fatal and nonfatal) myocardial infarction and stroke as outcomes if the numbers of nonfatal myocardial infarction and stroke could not be derived. Revascularization included any percutaneous coronary intervention or coronary artery bypass grafting reported in the clinical trials. The overall cardiovascular events were calculated as the aggregation of cardiovascular death, myocardial infarction, stroke, and revascularization.

We assessed the quality of the included studies using the Jadad scale,^12^ which measures the methodologic quality of randomized clinical trials on a scale of 0 to 5, with 0 indicating very low and 5 indicating rigorous quality. This scale assesses the risk of bias based on 3 domains: randomization (including mention of randomization and appropriate method of randomization), blinding (including mention of blinding and appropriate blinding), and consideration of all patients (ie, the outcomes of all patients in the clinical trial are known and, if no data are reported, the reason for missing data is stated). A study could be assigned a maximum of 2 points each for the domains of randomization and blinding and 1 point for the domain of consideration of all patients, for a possible maximum score of 5. Two of us (J.W. and A.J.K.) conducted the quality assessment and assigned quality scores (continuous measure) for each study. Studies scoring 3 or more points were deemed to have a low risk of bias, and studies scoring less than 3 points were deemed to have a high risk of bias.

### Data Synthesis and Analysis

The analysis was conducted from October 2017 to December 2019. We first conducted a pairwise meta-analysis of placebo-clinical trials to estimate the direct effect on reducing cardiovascular events reported for each agent compared with placebo. The risk difference (per 1000 persons) and the numbers needed to treat were calculated for each type of medication.

We conducted a frequentist network meta-analysis with random-effects models to estimate the aggregate reductions in cardiovascular events and revascularization for each type of antihypertension medication compared with placebo and with each other.^13^ We used Stata software, version 14.0 (StataCorp), for all analyses using the network command.^14^ This approach is an extension of the method proposed by DerSimonian and Laird,^15^ and the performance of this model has been satisfactory. The model contains a component of inconsistency variance, which is a source of variation in addition to between-study heterogeneity.^16^ We reported risk ratios (RRs) and corresponding 95% CIs, and we calculated a pooled RR and 95% CI for each intervention group separately from each placebo group.

For each medication class, we assessed heterogeneity across studies using the maximum likelihood method.^16^ We examined the magnitude of a common heterogeneity variance for the network (τ^2^) as an indicator of the extent of heterogeneity among included studies in terms of the range of expected treatment estimates (RRs and 95% CIs). Any τ^2^ values under 0.25 were considered acceptable heterogeneity; values between 0.25 and 1.0 represented moderately high heterogeneity; and values greater than 1.0 represented very high heterogeneity.

We assessed the general within-network inconsistency between direct effects (comparison between specific agents and placebo) and indirect comparisons (comparisons other than direct within each outcome) for each outcome using χ^2^ tests. If no general inconsistency was detected, the inconsistency between each of 2 agents was tested by calculating the differences in direct effects and indirect comparisons using their SEs.^17,18^ We considered evidence of inconsistency if *P* values were less than .05. We assessed potential publication bias by inspecting the symmetry of funnel plots for each outcome.^19^

We conducted metaregression analyses to examine the dose-response relationship between each within-treatment 10–mm Hg reduction in systolic blood pressure and 5–mm Hg reduction in diastolic blood pressure (regardless of intervention groups) over time and to assess the incidence of cardiovascular events (including overall and individual types of cardiovascular events, with the log RRs of cardiovascular events as dependent variables). The coefficient of metaregression was weighted by 1 divided by the sum of σ_i_^2^ and τ^2^, with σ_i_^2^ representing the SE of the estimated effect in the particular clinical trial and with τ^2^ representing the between-study variance.^20^ In addition, we reported rates of adverse effects, such as edema, headache, cough, and hypotension or dizziness, that are associated with antihypertension medications and placebo among studies with available information.

## Results

**Figure 1.**
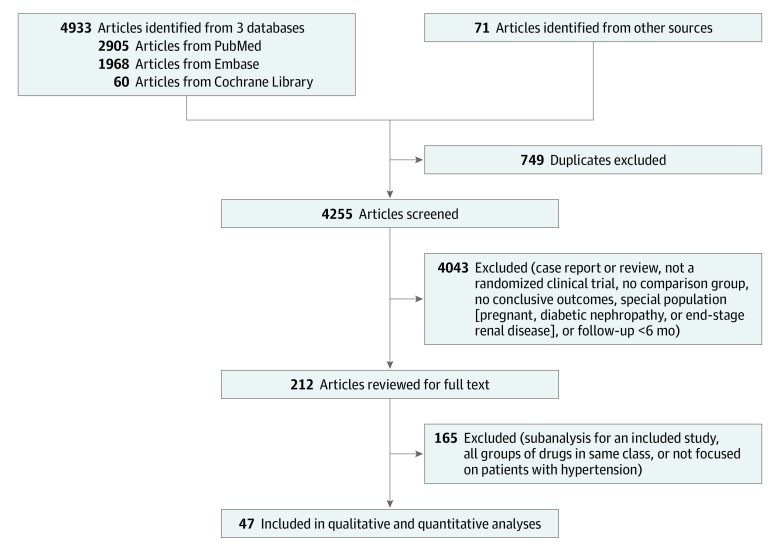
PRISMA Diagram of Literature Search and Selection Reasons for exclusion were not categorized by individual article because each article may have met multiple exclusion criteria.

Our systematic search yielded 4933 articles (Figure 1). We identified an additional 71 articles from bibliographies of relevant reports and reviews. In total, the systematic review and network meta-analysis included 47 published articles from 46 clinical trials, with 248 887 participants and 28 658 first-in-trial cardiovascular events. Owing to the small number of studies reporting different medication combination permutations and non–DH CCBs, we did not report data for fixed-dose antihypertension combination medications in the network meta-analysis.

**Figure 2.**
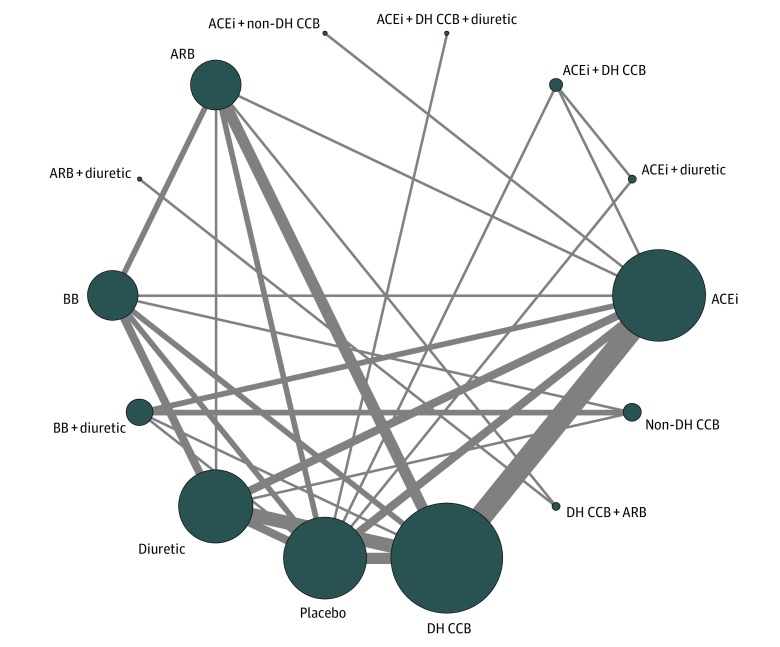
Network Plot of Antihypertension Medications ACEi indicates angiotensin-converting enzyme inhibitor; ARB, angiotensin receptor blocker; BB, β-blocker; DH CCB, dihydropyridine calcium channel blocker; and non–DH CCB, nondihydropyridine calcium channel blocker.

Figure 2 shows the network plot depicting the different antihypertension medication classes and comparisons tested. Among included studies, 15 tested ACE inhibitors, 23 tested DH CCBs, 4 tested non–DH CCBs, 8 tested β-blockers, 12 tested ARBs, and 13 tested thiazide diuretics. Four studies were from North America, 18 from Europe, 16 from Asia, 1 from Oceania, and 7 from multiple regions across continents.

The mean (SD) age of participants was 65.6 (5.8) years (range, 51.8-83.8 years), and 52.8% (range, 28.2%-100.0%) were men. The mean (SD) baseline systolic blood pressure was 161.3 (13.6) mm Hg (range, 129-195 mm Hg). A total of 14 studies included placebo, health education, or conventional education in their control groups, and 35 studies reported direct treatment comparisons (3 of which included placebo and 2 treatment agents). The mean (SD) follow-up time was 3.7 (1.5) years (range, 1.0-10.0 years). Across all included clinical trials with particular classes of antihypertension medications, the frequency of use for each type of antihypertension medication was similar between studies, with the participants’ mean age above and below 65 years. By region of study, ACE inhibitors, β-blockers, and diuretics were more frequently used in Europe, DH CCBs were more frequently used in Asia and Europe, and ARBs were more frequently used in Asia. The prevalence of baseline cardiovascular disease and diabetes was similar among participants using each class of antihypertension medications. The characteristics of all 46 included studies are available in eTable 1 in the Supplement.

For all 46 clinical trials included in the analysis, the mean (SD) overall systolic and diastolic blood pressure changes among those reported were −18.0 (8.7) mm Hg and −10.1 (4.7) mm Hg, respectively. The overall risk of cardiovascular events was 11.5%. Among the adverse effect profile, edema was prevalent among 0.5% of participants receiving ACE inhibitors, 17.1% of participants receiving DH CCBs or non–DH CCBs, 8.7% of participants receiving β-blockers, 15.1% of participants receiving ARBs, 9.9% of participants receiving diuretics, and 3.7% of participants receiving placebo. Cough was prevalent among 8.3% of participants receiving ACE inhibitors, 9.6% of participants receiving DH CCBs or non–DH CCBs, 4.1% of participants receiving β-blockers, 2.7% of participants receiving ARBs, 5.4% of participants receiving diuretics, and 16.1% of participants receiving placebo. Headache or hypotension was prevalent among 0.7% of participants receiving ACE inhibitors, 7.9% of participants receiving DH CCBs or non–DH CCBs, 1.4% of participants receiving β-blockers, 10.8% of participants receiving ARBs, 7.4% of participants receiving diuretics, and 8.8% of participants receiving placebo. Dizziness was prevalent among 1.7% of participants receiving ACE inhibitors, 7.5% of participants receiving DH CCBs or non–DH CCBs, 9.1% of participants receiving β-blockers, 14.8% of participants receiving ARBs, 9.0% of participants receiving diuretics, and 10.2% of participants receiving placebo.

### Comparative Efficacy 

An assessment of the absolute risk differences in cardiovascular events among placebo-clinical trials that compared antihypertension medications with placebo indicated that all 5 types of antihypertension medications were associated with larger decreases in cardiovascular events than placebo. Compared with placebo, DH CCBs were reported to reduce the risk of cardiovascular death by 7 cases per 1000 persons; ACE inhibitors and diuretics were reported to decrease myocardial infarction risk by 24 cases and 10 cases per 1000 persons, respectively; and DH CCBs and diuretics were reported to reduce stroke risk by 16 cases and 21 cases per 1000 persons, respectively (eTable 2 in the Supplement).

Based on these results, 143 people would need to be treated with DH CCBs to prevent 1 cardiovascular death. To prevent 1 myocardial infarction, 42 people would need to be treated with ACE inhibitors and 100 people with diuretics. To prevent 1 stroke, 63 people would need to be treated with DH CCBs and 48 people with diuretics.

### Network Meta-analysis 

In the network meta-analysis comparing placebo vs medication groups, for cardiovascular death, we noted that ACE inhibitors, DH CCBs, ARBs, and diuretics were all associated with 15% to 22% relative risk reductions. The specific relative risk reductions were as follows: for ACE inhibitors, 20% reduction (95% CI, 9%-30%; RR, 0.80; 95% CI, 0.70-0.91); for DH CCBs, 20% reduction (95% CI, 11%-29%; RR, 0.80; 95% CI, 0.71-0.89); for ARBs, 15% reduction (95% CI, 3%-26%; RR, 0.85; 95% CI, 0.74-0.97); and for diuretics, 22% reduction (95% CI, 12%-31%; RR, 0.78; 95% CI, 0.69-0.88).

For myocardial infarction, ACE inhibitors, DH CCBs, and β-blockers were associated with 20% to 28% relative risk reductions. The specific relative risk reductions were as follows: for ACE inhibitors, 28% reduction (95% CI, 12%-41%; RR, 0.72; 95% CI, 0.59-0.88); for DH CCBs, 21% reduction (95% CI, 4%-34%; RR, 0.79; 95% CI, 0.66-0.96); and for β-blockers, 20% reduction (95% CI, 1%-35%; RR, 0.80; 95% CI, 0.65-0.99).

For stroke, all classes of medications were associated with 19% to 39% relative risk reductions. The specific relative risk reductions were as follows: for ACE inhibitors, 34% reduction (95% CI, 20%-45%; RR, 0.66; 95% CI, 0.55-0.80); for DH CCBs, 39% reduction (95% CI, 30%-48%; RR, 0.61; 95% CI, 0.52-0.70); for β-blockers, 20% reduction (95% CI, 2%-33%; RR, 0.80; 95% CI, 0.67-0.98); for ARBs, 31% reduction (95% CI, 17%-42%; RR, 0.69; 95% CI, 0.58-0.83); and for diuretics, 37% reduction (95% CI, 24%-47%; RR, 0.63; 95% CI, 0.53-0.76).

**Figure 3.**
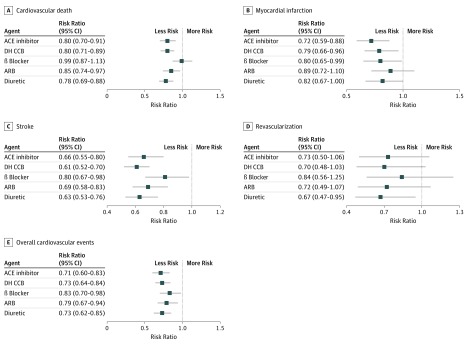
Network Meta-analysis Comparing Single Class of Antihypertension Medications With Placebo for Treatment of Cardiovascular Events ACE indicates angiotensin-converting enzyme; ARB, angiotensin receptor blocker; DH CCB, dihydropyridine calcium channel blocker; and error bars, 95% CI.

For revascularization, diuretics were associated with a 33% relative risk reduction (95% CI, 5%-53%; RR, 0.67; 95% CI, 0.47-0.95). For overall cardiovascular events, all classes of medications were each associated with a 17% to 29% relative risk reduction (Figure 3). The specific relative risk reductions were as follows: for ACE inhibitors, 29% reduction (95% CI, 17%-40%; RR, 0.71; 95% CI, 0.60-0.83); for DH CCBs, 27% reduction (95% CI, 16%-36%; RR, 0.73; 95% CI, 0.64-0.84); for β-blockers, 17% reduction (95% CI, 2%-30%; RR, 0.83; 95% CI, 0.70-0.98); for ARBs, 21% reduction (95% CI, 6%-33%; RR, 0.79; 95% CI, 0.67-0.94); and for diuretics, 27% reduction (95% CI, 15%-38%; RR, 0.73; 95% CI, 0.62-0.85).

These data indicate that, according to effect sizes, ACE inhibitors, DH CCBs, and diuretics were associated with similarly significant reductions in the risk of overall cardiovascular events and cardiovascular death. The greatest reductions in myocardial infarction risk were associated with ACE inhibitors, and DH CCBs and diuretics were associated with similarly significant reductions in the risk of stroke. Diuretics were associated with similarly significant reductions in the risk of revascularization. However, their 95% CIs overlapped.

### Consistency, Heterogeneity, Bias, and Sensitivity

In terms of the risk of bias assessment, we found that studies were of moderate quality (mean score, 4.0 of 5.0 possible points), in which randomization had a mean (SD) score of 1.8 (1.0) of 2.0 points, blinding had a mean (SD) score of 1.1 (0.5) of 2.0 points, and consideration of all patients had a mean (SD) score of 1.0 (1.0) of 1.0 point. No study was considered to have a high risk of bias. Visual examination of funnel plots indicated that no significant publication bias was present for cardiovascular death, myocardial infarction, stroke, revascularization, and overall cardiovascular events (eFigure 1 in the Supplement).

No general inconsistency of treatment effect on each outcome was found, with all *P* values greater than .05. By individual treatment, no significant inconsistency was detected among different classes of antihypertension medications (eTable 3 in the Supplement). No significant treatment effect heterogeneity was detected, as none of the τ^2^ values was equal to or greater than 0.25 (eFigure 2 in the Supplement).

In metaregressions of blood pressure reduction with cardiovascular events, blood pressure reduction was associated with reductions in cardiovascular events. In particular, each 10–mm Hg reduction in systolic blood pressure was significantly associated with a 13% lower risk of cardiovascular death (RR, 0.87; 95% CI, 0.77-0.99), a 17% lower risk of stroke (RR, 0.83; 95% CI, 0.72-0.97), and a 14% lower risk of overall cardiovascular events (RR, 0.86; 95% CI, 0.78-0.96). Each 5–mm Hg reduction in diastolic blood pressure was significantly associated with a 14% lower risk of cardiovascular death (RR, 0.86; 95% CI, 0.74-1.00), a 20% lower risk of stroke (RR, 0.80; 95% CI, 0.67-0.95), and a 16% lower risk of overall cardiovascular events (RR, 0.84; 95% CI, 0.74-0.96; eTable 4 in the Supplement).

## Discussion

From 46 randomized clinical trials that examined the effect of the most commonly used antihypertension medications on preventing cardiovascular events, pooled results showed that ACE inhibitors, DH CCBs, and diuretics were reported to be similarly effective in preventing cardiovascular death (approximately 20% reduction compared with placebo), stroke (approximately 35% reduction compared with placebo), and overall cardiovascular events (approximately 30% reduction compared with placebo). Angiotensin-converting enzyme inhibitors were reported to be the most effective in preventing myocardial infarction (approximately 30% reduction compared with placebo). Diuretics were reported to be the most effective in reducing revascularization (approximately 30% compared with placebo). Our study provides the most current evidence to date on the comparative efficacy of antihypertension medications reported to reduce cardiovascular events in randomized clinical trials. Furthermore, to our knowledge, this is the first meta-analysis to pool the results of studies that tested the efficacy of antihypertension medications in reducing the risk of revascularization.

Our results are consistent with those reported in previous meta-analyses of randomized clinical trials.^8,9^ Psaty et al^9^ found that low-dose diuretics were reported to be the most effective treatment for preventing the occurrence of cardiovascular mortality. Law et al^8^ indicated that for stroke, all antihypertension medication classes were reported to have similar risk reduction effects for a given reduction in blood pressure. Our findings further supported the evidence indicating that the reported differences of the effects on reducing cardiovascular events between medication classes are small. We also provided more up-to-date information regarding the reported efficacy of antihypertension medications in reducing cardiovascular events. A recent systematic multinational analysis, the Large-Scale Evidence Generation and Evaluation Across a Network of Databases for Hypertension (LEGEND-HTN) study,^21^ used observational data encompassing millions of patients and found that diuretics were associated with advantages in reducing various cardiovascular events compared with other classes of medications. This finding is different than that of our study, which indicated that diuretics had similar reported effectiveness in reducing cardiovascular events compared with ACE inhibitors and DH CCBs and that ACE inhibitors appeared to be most effective in reducing myocardial infarction. The difference in findings may be owing to the varied study designs (randomized clinical trials vs observational studies), and it is not clear to what extent the LEGEND-HTN study may be subject to selection bias.

Network meta-analysis allowed us to compare medication classes with placebo both directly (ie, among the included placebo-clinical trials) and indirectly (ie, among all of the included studies). The comparisons between medications and placebo indicated slight differences between direct and overall comparisons. The network comparisons were more precise (ie, they had narrower 95% CIs).

It is also worth noting that the association between decreasing blood pressure and reduced cardiovascular events was smaller than that obtained from observational studies.^22,23^ The reason may be that most participants in clinical trials, including those receiving placebo, were motivated to reduce their blood pressure and, in a number of cases, were also treated with other medications. In addition, participants’ motivation may have led them to engage in healthy lifestyle habits, such as choosing healthier diet patterns, exercising, and avoiding tobacco and alcohol use. Dietary modification can and should be a complementary effort in trying to reduce BP and many dietary patterns are supported by robust evidence.^27^ This finding also calls for future studies that examine the associations between antihypertension medications and lifestyle in preventing cardiovascular events among patients with hypertension. However, although the effect size for the association between decreasing blood pressure and reduced cardiovascular events was small, some of the differences were statistically significant.

### Limitations

Our study was limited by the relatively small number of studies included in the network meta-analysis, so we lacked the statistical power to conduct subgroup analyses to examine whether the association of antihypertension medications with reduced cardiovascular events could be the consequence of different factors (eg, age, sex, or baseline blood pressure level). Although the results from this analysis may serve as a source of reference, a comprehensive study based on demographic factors and comorbidities is needed to assess which class of antihypertension medication should be recommended for reducing adverse cardiovascular outcomes. The newly published American College of Cardiology and American Heart Association guidelines for hypertension identify a wider range of individuals with early hypertension and prehypertension, and our study can guide first-line medication choices.^24^ In addition, the 46 clinical trials we studied were mostly performed in North America, western Europe, and East Asia, and more data are needed regarding patients in South Asia and Africa, which cumulatively compose a large proportion of the world’s population.

Another important limitation of our study is that we did not include results for combinations of antihypertension medications because there were too few studies with data for each permutation of combinations. However, this lack of studies suggests that the efficacy of several groups of combinations, such as β-blockers and diuretics, will need to be studied more frequently in the future. Recent findings have indicated that treatment with low doses of 3 antihypertensive medications is associated with an increased proportion of patients who achieved target blood pressure compared with standard care,^25^ and quarter-dose therapies with combined medications were reported to be more effective, with fewer adverse events, in reducing blood pressure compared with standard monotherapy.^26^

## Conclusions

The present network meta-analysis indicated that major first-line antihypertension medications, including ACE inhibitors, DH CCBs, β-blockers, ARBs, and diuretics, were all reported to be effective in reducing cardiovascular events compared with placebo. Furthermore, ACE inhibitors, DH CCBs, and diuretics appeared to be similarly effective in reducing cardiovascular deaths, stroke, and overall cardiovascular events. Compared with other antihypertension medications, ACE inhibitors appeared to be the medications of choice to prevent myocardial infarction, and diuretics appeared to be the optimal choice to reduce revascularization. The differences between medication classes were generally small in terms of their associations with reducing cardiovascular events. Future studies should compare the effectiveness of multiple antihypertension medications in combination with individual antihypertension medications in reducing cardiovascular events.

## Notes

Supplement. **eMethods.** Search Terms **eTable 1.** Cardiovascular Events and Outcomes by Randomized Treatment **eTable 2.** Absolute Risk Differences of Cardiovascular Events Comparing Each Type of Antihypertension Medication to Placebo From Network Meta-analysis **eTable 3.** Inconsistency Test Between Direct and Indirect Treatment Comparisons in Mixed Treatment Comparisons **eTable 4.** Bivariate Meta-regression on the 10 mm Hg Reduction of Blood Pressure and Change of Cardiovascular Events **eFigure 1.** Funnel Plots of Studies of Antihypertension Medications and Effects of Prevention on Cardiovascular Death, Myocardial Infarction, Stroke, Revascularization, and Overall Cardiovascular Events **eFigure 2.** Inconsistency Plots of Studies of Antihypertension Medications and Effects of Prevention on Cardiovascular Death, Myocardial Infarction, Stroke, Revascularization, and Overall Cardiovascular Events **eReferences.**

## References

[1] Forouzanfar MH, Alexander L, Anderson HR, ; GBD 2013 Risk Factors Collaborators Global, regional, and national comparative risk assessment of 79 behavioural, environmental and occupational, and metabolic risks or clusters of risks in 188 countries, 1990-2013: a systematic analysis for the Global Burden of Disease Study 2013. Lancet. 2015;386(10010):-. doi:10.1016/S0140-6736(15)00128-2 26364544 PMC4685753

[2] World Health Organization Cardiovascular diseases (CVDs). World Health Organization website. https://www.who.int/mediacentre/factsheets/fs317/en/. Published May 17, 2017. Accessed July 1, 2017.

[3] Franklin SS, Wong ND Hypertension and cardiovascular disease: contributions of the Framingham heart study. Glob Heart. 2013;8(1):49-57. doi:10.1016/j.gheart.2012.12.004 25690263

[4] Bloch MJ Worldwide prevalence of hypertension exceeds 1.3 billion. J Am Soc Hypertens. 2016;10(10):753-754. doi:10.1016/j.jash.2016.08.006 27660007

[5] Khera R, Lu Y, Lu J, Impact of 2017 ACC/AHA guidelines on prevalence of hypertension and eligibility for antihypertensive treatment in United States and China: nationally representative cross sectional study. BMJ. 2018;362:k2357. doi:10.1136/bmj.k2357 29997129 PMC6039831

[6] Venkateshmurthy NS, Geldsetzer P, Jaacks LM, Prabhakaran D Implications of the New American College of Cardiology guidelines for hypertension prevalence in India. JAMA Intern Med. 2018;178(10):1416-1418. doi:10.1001/jamainternmed.2018.3511 30083722 PMC6233754

[7] Ettehad D, Emdin CA, Kiran A, Blood pressure lowering for prevention of cardiovascular disease and death: a systematic review and meta-analysis. Lancet. 2016;387(10022):957-967. doi:10.1016/S0140-6736(15)01225-8 26724178

[8] Law MR, Morris JK, Wald NJ Use of blood pressure lowering drugs in the prevention of cardiovascular disease: meta-analysis of 147 randomised trials in the context of expectations from prospective epidemiological studies. BMJ. 2009;338:b1665. doi:10.1136/bmj.b1665 19454737 PMC2684577

[9] Psaty BM, Lumley T, Furberg CD, Health outcomes associated with various antihypertensive therapies used as first-line agents: a network meta-analysis. JAMA. 2003;289(19):2534-2544. doi:10.1001/jama.289.19.2534 12759325

[10] Whelton PK, Carey RM, Aronow WS, 2017 ACC/AHA/AAPA/ABC/ACPM/AGS/APhA/ASH/ASPC/NMA/PCNA guideline for the prevention, detection, evaluation, and management of high blood pressure in adults: a report of the American College of Cardiology/American Heart Association Task Force on Clinical Practice Guidelines. Hypertension. 2018;71(6):1269-1324. doi:10.1161/HYP.0000000000000066 29133354

[11] Moher D, Liberati A, Tetzlaff J, Altman DG; PRISMA Group Preferred reporting items for systematic reviews and meta-analyses: the PRISMA statement. BMJ. 2009;339:b2535. doi:10.1136/bmj.b2535 19622551 PMC2714657

[12] Jadad AR, Moore RA, Carroll D, Assessing the quality of reports of randomized clinical trials: is blinding necessary? Control Clin Trials. 1996;17(1):1-12. doi:10.1016/0197-2456(95)00134-4 8721797

[13] Rucker G, Schwarzer G Ranking treatments in frequentist network meta-analysis works without resampling methods. BMC Med Res Methodol. 2015;15:58. doi:10.1186/s12874-015-0060-8 26227148 PMC4521472

[14] Chaimani A, Higgins JPT, Mavridis D, Spyridonos P, Salanti G Graphical tools for network meta-analysis in STATA. PLoS One. 2013;8(10):e76654. doi:10.1371/journal.pone.0076654 24098547 PMC3789683

[15] Jackson D, Law M, Barrett JK, Extending DerSimonian and Laird’s methodology to perform network meta-analyses with random inconsistency effects. Stat Med. 2016;35(6):819-839. doi:10.1002/sim.6752 26423209 PMC4973704

[16] Law M, Jackson D, Turner R, Rhodes K, Viechtbauer W Two new methods to fit models for network meta-analysis with random inconsistency effects. BMC Med Res Methodol. 2016;16:87. doi:10.1186/s12874-016-0184-5 27465416 PMC4964019

[17] Higgins JP, Jackson D, Barrett JK, Lu G, Ades AE, White IR Consistency and inconsistency in network meta-analysis: concepts and models for multi-arm studies. Res Synth Methods. 2012;3(2):98-110. doi:10.1002/jrsm.1044 26062084 PMC4433772

[18] Shim S, Yoon B-H, Shin I-S, Bae J-M Network meta-analysis: application and practice using Stata. Epidemiol Health. 2017;39:e2017047. doi:10.4178/epih.e2017047 29092392 PMC5733388

[19] Sedgwick P Meta-analyses: how to read a funnel plot. BMJ. 2013;346:f1342. doi:10.1136/bmj.f1342 26377337

[20] Harbord RM, Higgins JPT Meta-regression in Stata. Stata J. 2008;8(4):493-519. doi:10.1177/1536867X0800800403

[21] Suchard MA, Schuemie MJ, Krumholz HM, Comprehensive comparative effectiveness and safety of first-line antihypertensive drug classes: a systematic, multinational, large-scale analysis. Lancet. 2019;394(10211):1816-1826. doi:10.1016/S0140-6736(19)32317-7 31668726 PMC6924620

[22] MacMahon S, Peto R, Collins R, Blood pressure, stroke, and coronary heart disease: part 1, prolonged differences in blood pressure: prospective observational studies corrected for the regression dilution bias. Lancet. 1990;335(8692):765-774. doi:10.1016/0140-6736(90)90878-9 1969518

[23] Asia Pacific Cohort Studies Collaboration Blood pressure and cardiovascular disease in the Asia Pacific region. J Hypertens. 2003;21(4):707-716. doi:10.1097/00004872-200304000-00013 12658016

[24] Carey RM, Whelton PK; 2017 ACC/AHA Hypertension Guideline Writing Committee Prevention, detection, evaluation, and management of high blood pressure in adults: synopsis of the 2017 American College of Cardiology/American Heart Association hypertension guideline. Ann Intern Med. 2018;168(5):351-358. doi:10.7326/M17-3203 29357392

[25] Webster R, Salam A, de Silva HA, ; TRIUMPH Study Group Fixed low-dose triple combination antihypertensive medication vs usual care for blood pressure control in patients with mild to moderate hypertension in Sri Lanka: a randomized clinical trial. JAMA. 2018;320(6):566-579. doi:10.1001/jama.2018.10359 30120478 PMC6583010

[26] Bennett A, Chow CK, Chou M, Efficacy and safety of quarter-dose blood pressure–lowering agents: a systematic review and meta-analysis of randomized controlled trials. Hypertension. 2017;70(1):85-93. doi:10.1161/HYPERTENSIONAHA.117.09202 28584013

[27] Gay HC, Rao SG, Vaccarino V, Ali MK Effects of different dietary interventions on blood pressure: systematic review and meta-analysis of randomized controlled trials. Hypertension. 2016;67(4):733-739. doi:10.1161/HYPERTENSIONAHA.115.06853 26902492

