# Supplementary material for: Comparison of Cardiovascular Events Among Users of Different Classes of Antihypertension Medications: A Systematic Review and Network Meta-analysis
Source: JAMA Netw Open. 2020 Feb 21;3(2):e1921618. doi: 10.1001/jamanetworkopen.2019.21618 (PMC7043193; doi:10.1001/jamanetworkopen.2019.21618)
Supplement: Supplement. — eMethods. Search Terms eTable 1. Cardiovascular Events and Outcomes by Randomized Treatment eTable 2. Absolute Risk Differences of Cardiovascular Events Comparing Each Type of Antihypertension Medication to Placebo From Network Meta-analysis eTable 3. Inconsistency Test Between Direct and Indirect Treatment Comparisons in Mixed Treatment Comparisons eTable 4. Bivariate Meta-regression on the 10 mm Hg Reduction of Blood Pressure and Change of Cardiovascular Events eFigure 1. Funnel Plots of Studies of Antihypertension Medications and Effects of Prevention on Cardiovascular Death, Myocardial Infarction, Stroke, Revascularization, and Overall Cardiovascular Events eFigure 2. Inconsistency Plots of Studies of Antihypertension Medications and Effects of Prevention on Cardiovascular Death, Myocardial Infarction, Stroke, Revascularization, and Overall Cardiovascular Events eReferences. [file jamanetwopen-3-e1921618-s001.pdf]

Supplementary Online Content

Wei J, Galaviz KI, Kowalski AJ, Magee MJ, Haw JS, Narayan KMV, Ali MK. Comparison of cardiovascular events among users of different classes of antihypertension drugs: a systematic review and network meta-analysis. *JAMA Netw Open.* Published February 21, 2020. doi:10.1001/jamanetworkopen.2019.21618

eMethods. Search Terms

eTable 1. Cardiovascular Events and Outcomes by Randomized Treatment

eTable 2. Absolute Risk Differences of Cardiovascular Events Comparing Each Type of Blood Pressure–Lowering Drug to Placebo From Network Meta-analysis

eTable 3. Inconsistency Test Between Direct and Indirect Treatment Comparisons in Mixed Treatment Comparisons

eTable 4. Bivariate Meta-regression on the 10 mm Hg Reduction of Blood Pressure and Change of Cardiovascular Events

eFigure 1. Funnel Plots of Studies of Blood Pressure–Lowering Drugs and Effects of Prevention on Cardiovascular Death, Myocardial Infarction, Stroke, Revascularization, and Overall Cardiovascular Events

eFigure 2. Inconsistency Plots of Studies of Blood Pressure–Lowering Drugs and Effects of Prevention on Cardiovascular Death, Myocardial Infarction, Stroke, Revascularization, and Overall Cardiovascular Events

eReferences.

This supplementary material has been provided by the authors to give readers additional information about their work.

© 2020 Wei J et al. *JAMA Netw Open*. All rights reserved.

eMethods. Search Terms

("hypertension"[MeSH Terms] OR "hypertension"[All Fields]) AND (("antihypertensive agents"[Pharmacological Action] OR "antihypertensive agents"[MeSH Terms] OR "antihypertensive agents"[All Fields]) OR ("sodium chloride symporter inhibitors"[Pharmacological Action] OR "sodium chloride symporter inhibitors"[MeSH Terms] OR "sodium chloride symporter inhibitors"[All Fields]) OR "thiazide diuretics"[All Fields] OR ("adrenergic beta-antagonists"[Pharmacological Action] OR "adrenergic beta-antagonists"[MeSH Terms] OR "adrenergic beta-antagonists"[All Fields]) OR ("angiotensin-converting enzyme inhibitors"[Pharmacological Action] OR "angiotensin-converting enzyme inhibitors"[MeSH Terms] OR "angiotensin-converting enzyme inhibitors"[All Fields]) OR ("angiotensin receptor antagonists"[Pharmacological Action] OR "angiotensin receptor antagonists"[MeSH Terms] OR "angiotensin receptor antagonists"[All Fields]) OR ("calcium channel blockers"[Pharmacological Action] OR "calcium channel blockers"[MeSH Terms] OR "calcium channel blockers"[All Fields]) OR ("vasodilator agents"[Pharmacological Action] OR "vasodilator agents"[MeSH Terms] OR "vasodilator agents"[All Fields])) AND ((Controlled Clinical Trial[ptyp] OR Randomized Controlled Trial[ptyp]) AND "humans"[MeSH Terms] AND "adult"[MeSH Terms]) AND ("1990/01/01"[PDAT] : "2030/12/31"[PDAT])

© 2020 Wei J et al. *JAMA Netw Open*. All rights reserved.

eTable 1. Cardiovascular Events and Outcomes by Randomized Treatment

Study	Sample Size	Follow-Up Time	Treatment	Cardiovascular Events				Age	Male, %	Region
				Death	MI	Stroke	CABG/PCI			
DEMAND, 2011[1]	126	3 years	ACEi/DH CCB*	0	-	-	-	60.2	61.9	Italy Slovenia
	127		ACEi	0	-	-	-	61.9	64.6	
	127		Placebo	2	-	-	-	60.4	69.3	
FEVER, 2005[2]	4841	40 months	DH CCB	73	-	177	4	61.5	61.8	China
	4870		Placebo	101	-	251	11	61.5	60.5	
MRC, 1992[3]	1081	5.8 years	Diuretics	66	-	45	-	70	42.0	UK
	1102		BB	95	-	56	-	70	41.4	
	2213		Placebo	180	-	134	-	70	41.8	
SHEP, 1991[4]	2365	4.5 years	Diuretics	90	50	96	49	71.6	43.7	US
	2371		Placebo	112	74	149	69	71.5	42.7	
STOP, 1991[5]	812	25 months	BB/Diuretics	36	19	26	-	75.6	37	Sweden
	815		Placebo	63	22	41	-	75.7	37	
Syst-China, 1998[6]	1253	2 years	DH CCB	33	4	35	-	66.4	63.6	China
	1141		Placebo	44	4	41	-	66.7	65	
Syst-Eur, 1997[7]	2398	2 years	DH CCB	59	26	34	-	70.3	32.5	Europe
	2297		Placebo	77	31	57	-	70.2	33.8	
Sun, Ming, 1997[8]	1040	10 years	DH CCB	-	-	37	-	51.8	53.0	China
	1040		Placebo	-	-	79	-			
Sun, Z. Q, 2010[9]	2530	15 months	ACEi/Diuretics/DH CCB	-	-	24	-	56.4	43.8	China
	2454		Education	-	-	52	-	56.2	41.8	
HYVET Pilot, 2003[10]	426	13 months	Diuretics	23	-	-	-	83.8	37.1	Europe
	431		ACEi	22	-	-	-	83.7	36.0	
	426		Standard	19	-	-	-	83.8	36.6	
UKPDS, 1998[11,12]	400	9 years	ACEi	-	61	21	-	56.3	51.0	UK
	358		BB	-	46	17	-	56	57.0	
	390		Standard	-	69	34	-	56.5	58.0	
ABCD, 1998[13]	235	67 months	DH CCB	10	22	11	-	57.2	68.1	US
	235		ACEi	5	5	7	-	57.7	66.8	
ACCOMPLISH, 2008[14]	5744	3 years	ACE/DH CCB	107	125	112	334	68.4	60.0	US Europe
	5762		ACE/Diuretics	134	159	133	386	68.3	61.0	
ALLHAT, 2002[15]	15255	4.9 years	Diuretics	992	-	675	1113	66.9	53.0	US
	9048		DH CCB	592	-	377	725	66.9	52.7	
	9054		ACEi	609	-	457	718	66.9	53.8	
ASCOT, 2005[16]	9639	5.5 years	DH CCB	263	429	327	167	63	77.0	Europe

© 2020 Wei J et al. *JAMA Netw Open*. All rights reserved.

	9618		BB	342	474	422	214	63	77.0	

BENEDICT-B, 2011[17]	138	4.5 years	ACEi/Non-DH CCB	2	2	4	6	62.3	75.4	Italy
	143		ACEi	7	1	4	7	62.4	72.0	
COLM, 2014[18]	2568	3.3 years	DH CCB/ARB	13	9	60	-	73.6	51.5	Japan
	2573		Diuretics/ARB	18	16	62	-	73.6	51.7	
CONVINCE, 2003[19]	8179	3 years	Non-DH CCB	152	133	133	-	65.6	43.8	Multiple
	8297		BB/Diuretics	143	128	166	-	65.6	46.2	
ELSA, 2002[20]	1157	4 years	BB	8	17	14	-	55.9	55.4	Europe
	1177		DH CCB	4	18	9	-	56.1	54.2	
FACET, 1998[21]	189	3.5 years	ACEi	-	10	4	7	62.8	63.5	Italy
	191		DH CCB	-	13	10	8	63.3	55.5	
GLANT, 1995[22]	980	1 year	ACEi	1	1	5	-	60	44.4	Japan
	956		DH CCB	0	0	11	-	60	44.0	
INSIGHT, 2000[23]	3157	51 months	DH CCB	60	61	55	-	65	46.1	Europe Israel
	3164		Diuretics	52	56	63	-		46.6	
INVEST, 2003[24]	11267	2 years	Non-DH CCB	431	151	131	280	66	48.1	Multiple
	11309		BB	431	153	148	275	66.1	47.7	
J-MIND, 2001[25]	228	2 years	DH CCB	-	1	5	-	60.2	48.7	Japan
	208		ACEi	-	1	2	-	59.9	52.4	
JMIC-B, 2003[26]	828	3 years	DH CCB	6	16	16	81	65	67.6	Japan
	822		ACEi	6	13	16	75	64	70.0	
LIFE, 2002[27]	4605	4.8 years	ARB	204	198	232	261	66.9	46.0	US Europe
	4508		BB	234	188	309	284	66.9	46.0	
MAPHY, 1991[28]	1609	5 years	BB	29	82	21	6	40 to 64	100	Europe
	1625		Diuretics	34	109	18	8		100	
MIDAS, 1996[29]	442	3 years	DH CCB	3	6	6	11	58.2	79.9	US
	441		Diuretics	3	5	3	10	58.7	75.7	
MOSES, 2005[30]	681	2.5 years	ARB	-	-	236	-	67.7	53.6	Germany Austria
	671		DH CCB	-	-	134	-	68.1	54.8	
NAGOYA HEART, 2012[31]	575	3.2 years	ARB	-	7	13	29	63	66.0	Japan
	575		DH CCB	-	3	16	26	63	66.0	
NICS-EH, 1999[32]	204	5 years	DH CCB	2	2	1	-	69.7	40.2	Japan
	210		Diuretics	0	2	0	-	69.9	26.2	
NORDIL, 2000[33]	5410	4.5 years	Non-DH CCB	-	183	159	-	60.5	48.5	Sweden Norway
	5471		Diuretics/BB	-	157	196	-	60.3	48.7	
OSCAR, 2012[34]	578	3 years	ARB	-	-	24	-	73.6	43.9	Japan
	586		ARB/DH CCB	-	-	15	-	73.6	44.5	
PATE, 2000[35]	699	3 years	ACEi	-	1	11	-	70	38.3	Japan
	1049		DH CCB	-	5	17	-	69	43.3	

© 2020 Wei J et al. *JAMA Netw Open*. All rights reserved.

SHELL, 2003[36]	940	32 months	Diuretics	13	14	38	4	72.4	37.8	Italy
	942		DH CCB	16	12	37	2	72.3	39.6	
STOP-2, 1999[37]	2213	4 years	Diuretics/BB	221	154	237	-	76	32.0	Sweden
	2205		ACEi	226	139	215	-	76.1	33.7	
	2196		DH CCB	212	179	207	-	75.9	34.0	
VALUE, 2004[38]	7649	4.2 years	ARB	304	369	322	-	67.2	57.6	Multiple
	7596		DH CCB	304	313	281	-	67.3	57.5	
VART, 2011[39]	510	3.4 years	ARB	0	2	10	-	60	56.9	Japan
	511		DH CCB	0	1	10	-	60	57.5	
VHAS, 1997[40]	707	2 years	Diuretics	4	5	4	3	53.9	50.1	Italy
	707		Non-DH CCB	5	5	3	4	54.5	47.8	
SANBPS, 2003[41]	3044	4.1 years	ACEi	84	50	91	-	72	50	Australia
	3039		Diuretics	82	71	94	-	71.9	48	
CTHPCETG, 2011[42]	1110	3.6 years	ARB	-	-	17	-	63	51.0	Japan
	1089		BB	-	-	27	-	63.2	50.5	
	1094		Diuretics	-	-	12	-	63.1	50.5	
E-COST, 2005[43]	1053	3.1 years	ARB	0	10	47	-	-	44.5	Japan
	995		Conventional	0	23	77	-	-	51.8	
HYVET, 2014[44]	1922	1.8 years	ACE/Diuretics	88	9	23	-	83.5	39.5	Multiple
	1900		Placebo	110	11	28	-			
CAPPP, 1999[45]	5492	6.1 years	ACEi	70	137	173	-	52.4	54.9	Sweden
	5493		BB/Diuretics	81	128	127	-	52.7	52.0	
SCOPE, 2003[46]	2477	3.7 years	ARB	145	54	68	-	76.4	35.2	Europe
	2460		Placebo	152	47	93	-	76.4	35.8	
HIJ-CREATE, 2009[47]	1024	4.2 years	ARB	28	29	45	256	65.0	81.8	Japan
	1025		ACEi	25	26	49	271	64.5	78.6	

Abbreviations: ACEi, angiotensin-converting enzyme inhibitor; DH CCB, dihydropyridine calcium channel blocker; nDH CCB: BB, beta blocker; ARB, angiotensin receptor blocker; MI, myocardial infarction; CABG, coronary artery bypass surgery; PCI, percutaneous coronary intervention.

© 2020 Wei J et al. *JAMA Netw Open*. All rights reserved.

eTable 2. Absolute Risk Differences of Cardiovascular Events Comparing Each Type of Blood Pressure–Lowering Drug to Placebo From Network Meta-analysis

Drug	Cardiovascular Death	Myocardial Infarction	Stroke	Revascularization	Overall
ACEi vs. placebo	-0.005 (-0.027, 0.016)	**-0.024 (-0.036, -0.011)**	-0.035 (-0.070, 0.001)	**-**	-0.037 (-0.082, 0.009)
DH CCB vs. placebo	**-0.007 (-0.011, -0.003)**	-0.001 (-0.005, 0.003)	**-0.016 (-0.025, -0.006)**	-0.001 (-0.003, 0.0001)	-0.013 (-0.031, 0.005)
BB vs. placebo	0.005 (-0.015, 0.025)	-0.048 (-0.100, 0.003)	-0.020 (-0.048, 0.008)	-	-0.042 (-0.123, 0.039)
ARB vs. placebo	-0.000 (-0.002, 0.002)	-0.005 (-0.021, 0.011)	-0.020 (-0.041, 0.002)	-	-0.028 (-0.063, 0.007)
Diuretics vs. placebo	-0.010 (-0.022, 0.003)	**-0.010 (-0.019, -0.001)**	**-0.021 (-0.031, -0.011)**	-0.008 (-0.017, 0.0005)	-0.028 (-0.061, 0.005)

Abbreviations: ACEi, angiotensin-converting enzyme inhibitor; DH CCB, dihydropyridine calcium channel blocker; BB, beta blocker; ARB, angiotensin receptor blocker.

© 2020 Wei J et al. *JAMA Netw Open*. All rights reserved.

eTable 3. Inconsistency Test Between Direct and Indirect Treatment Comparisons in Mixed Treatment Comparisons

**Cardiovascular Mortality**							
**Side**	**Direct**		**Indirect**		**Difference**		**p>z**
	**Coefficient**	**SE**	**Coefficient**	**SE**	**Coefficient**	**SE**	
ACEi placebo	0.04	0.30	-0.23	0.07	0.28	0.31	0.37
DH CCB placebo	-0.33	0.10	-0.18	0.07	-0.15	0.12	0.22
BB placebo	0.05	0.12	-0.03	0.08	0.08	0.15	0.57
ARB placebo	-0.05	0.11	-0.23	0.09	0.18	0.14	0.20
Diuretics placebo	-0.20	0.09	-0.30	0.08	0.10	0.13	0.42
**Myocardial Infarction**							
**Side**	**Direct**		**Indirect**		**Difference**		**p>z**
	Coefficient	SE	Coefficient	SE	Coefficient	SE	
ACEi placebo	-0.15	0.18	-0.41	0.13	0.26	0.22	0.24
DH CCB placebo	-0.20	0.26	-0.23	0.11	-0.03	0.28	0.91
BB placebo	-0.29	0.19	-0.19	0.13	-0.10	0.23	0.67
ARB placebo	-0.11	0.19	-0.12	0.13	0.01	0.23	0.95
Diuretics placebo	-0.39	0.20	-0.12	0.12	-0.27	0.23	0.25
**Stroke**							
**Side**	**Direct**		**Indirect**		**Difference**		**p>z**
	**Coefficient**	**SE**	**Coefficient**	**SE**	**Coefficient**	**SE**	
ACEi placebo	-0.54	0.30	-0.40	0.10	-0.15	0.32	0.56
DH CCB placebo	-0.46	0.11	-0.54	0.11	0.09	0.15	0.57
BB placebo	-0.29	0.17	-0.18	0.12	-0.12	0.21	0.58
ARB placebo	-0.43	0.15	-0.33	0.11	-0.09	0.19	0.63
Diuretics placebo	-0.41	0.14	-0.50	0.12	0.09	0.19	0.64
**Revascularization**							
**Side**	**Direct**		**Indirect**		**Difference**		**p>z**
	**Coefficient**	**SE**	**Coefficient**	**SE**	**Coefficient**	**SE**	
DH CCB placebo	-1.01	0.58	-0.27	0.20	-0.73	0.62	0.24
Diuretics placebo	-0.34	0.19	-1.07	0.59	0.73	0.62	0.24
**Overall Cardiovascular Events**							
**Side**	**Direct**		**Indirect**		**Difference**		**p>z**
	**Coefficient**	**SE**	**Coefficient**	**SE**	**Coefficient**	**SE**	
ACEi placebo	-0.18	0.18	-0.40	0.10	0.22	0.21	0.30
DH CCB placebo	-0.31	0.11	-0.31	0.10	-0.002	0.15	0.99
BB placebo	-0.19	0.15	-0.18	0.11	-0.01	0.18	0.97
ARB placebo	-0.30	0.15	-0.19	0.11	-0.11	0.18	0.56
Diuretics placebo	-0.26	0.13	-0.36	0.11	-0.10	0.17	0.56

Abbreviations: ACEi, angiotensin-converting enzyme inhibitor; DH CCB, dihydropyridine calcium channel blocker; BB, beta blocker; ARB, angiotensin receptor blocker.

© 2020 Wei J et al. *JAMA Netw Open*. All rights reserved.

eTable 4. Bivariate Meta-regression on the 10 mm Hg Reduction of Blood Pressure and Change of Cardiovascular Events

**10 mm Hg Systolic Blood Pressure Reduction**	
**Cardiovascular Events**	**RR (95% CI)**
CVD mortality	**0.87 (0.77, 0.99)**
Myocardial infarction	0.95 (0.77, 1.17)
Stroke	**0.83 (0.72, 0.97)**
Revascularization	0.75 (0.50, 1.13)
Overall	**0.86 (0.78, 0.96)**
**5 mm Hg Diastolic Blood Pressure Reduction**	
**Cardiovascular Events**	**RR (95% CI)**
CVD mortality	**0.86 (0.74, 1.00)**
Myocardial infarction	0.95 (0.75, 1.21)
Stroke	**0.80 (0.67, 0.95)**
Revascularization	0.68 (0.42, 1.09)
Overall	**0.84 (0.74, 0.96)**

Abbreviation: RR, risk ratio.

© 2020 Wei J et al. *JAMA Netw Open*. All rights reserved.

eFigure 1. Funnel Plots of Studies of Blood Pressure–Lowering Drugs and Effects of Prevention on Cardiovascular Death, Myocardial Infarction, Stroke, Revascularization, and Overall Cardiovascular Events

A. Cardiovascular death

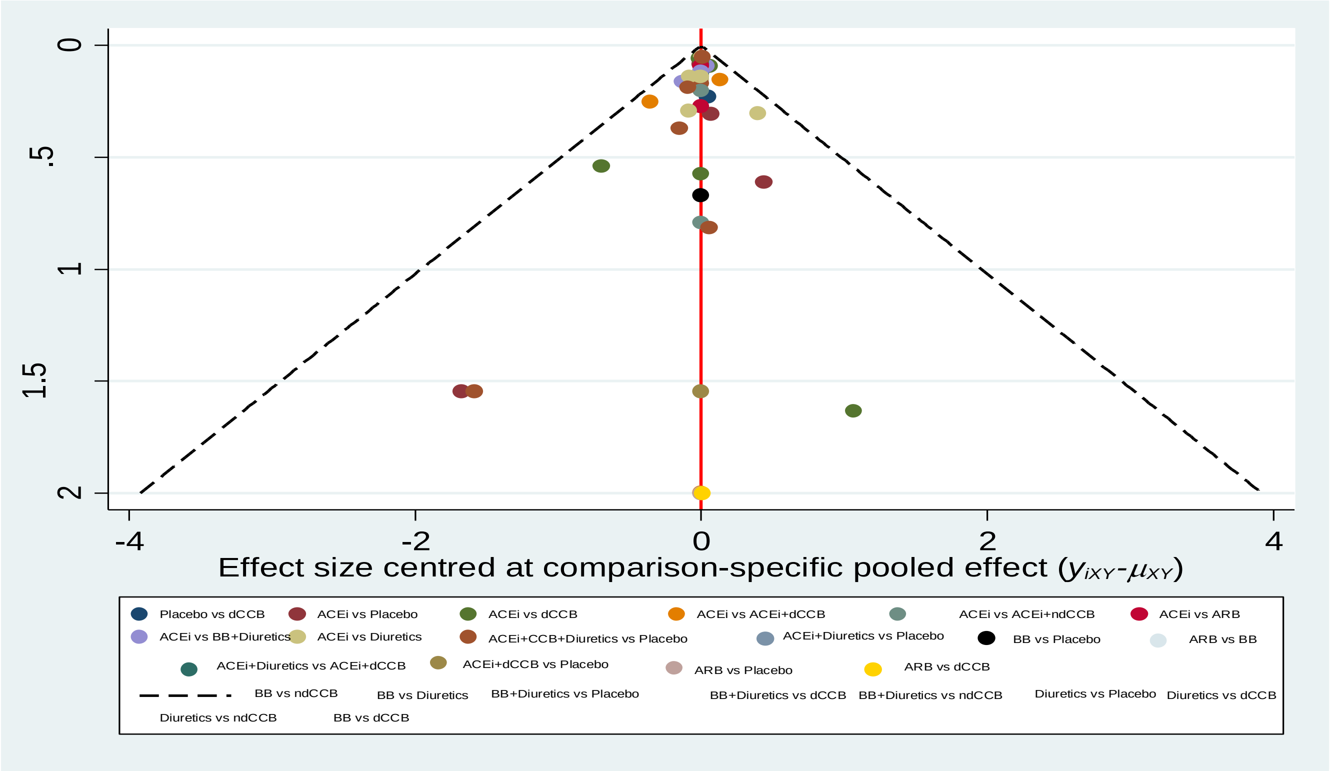

B. Myocardial infarction

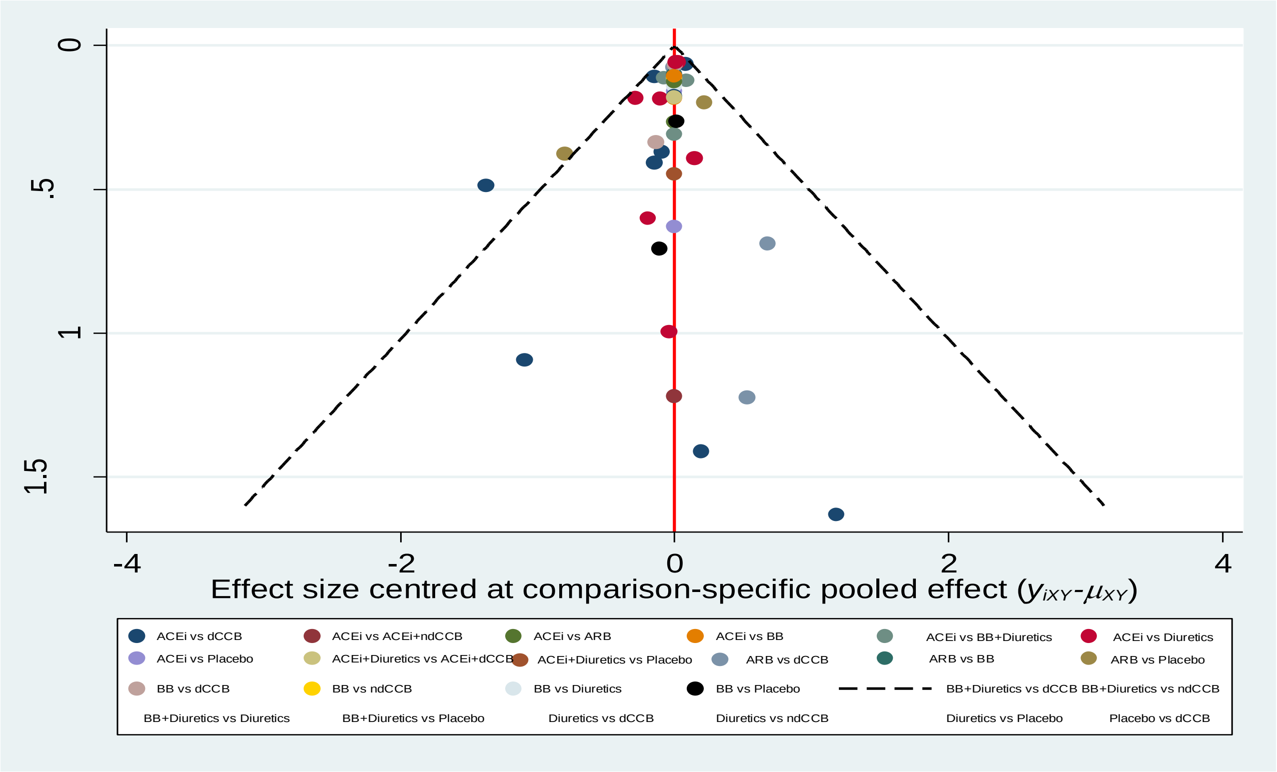

© 2020 Wei J et al. *JAMA Netw Open*. All rights reserved.

C. Stroke

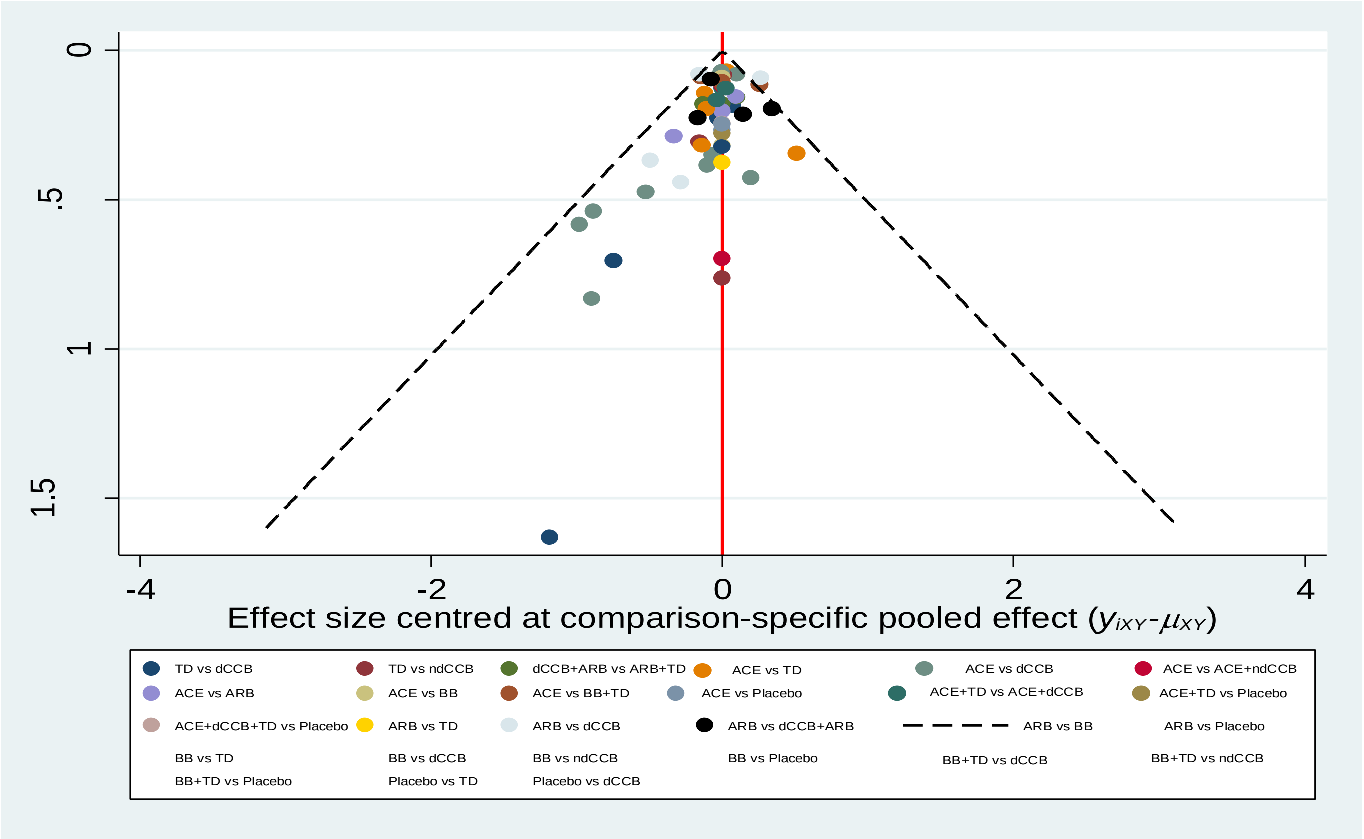

D. Revascularization

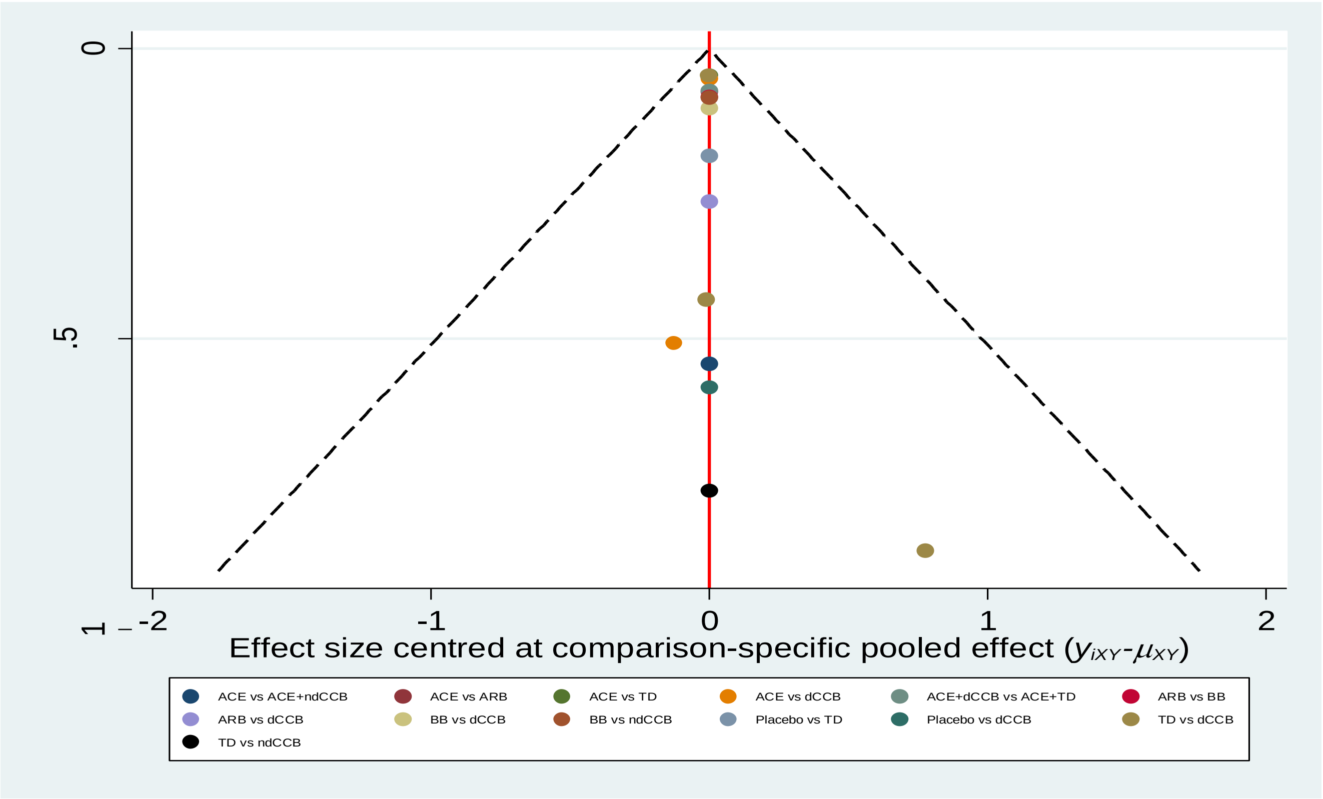

© 2020 Wei J et al. *JAMA Netw Open*. All rights reserved.

E. Overall cardiovascular events

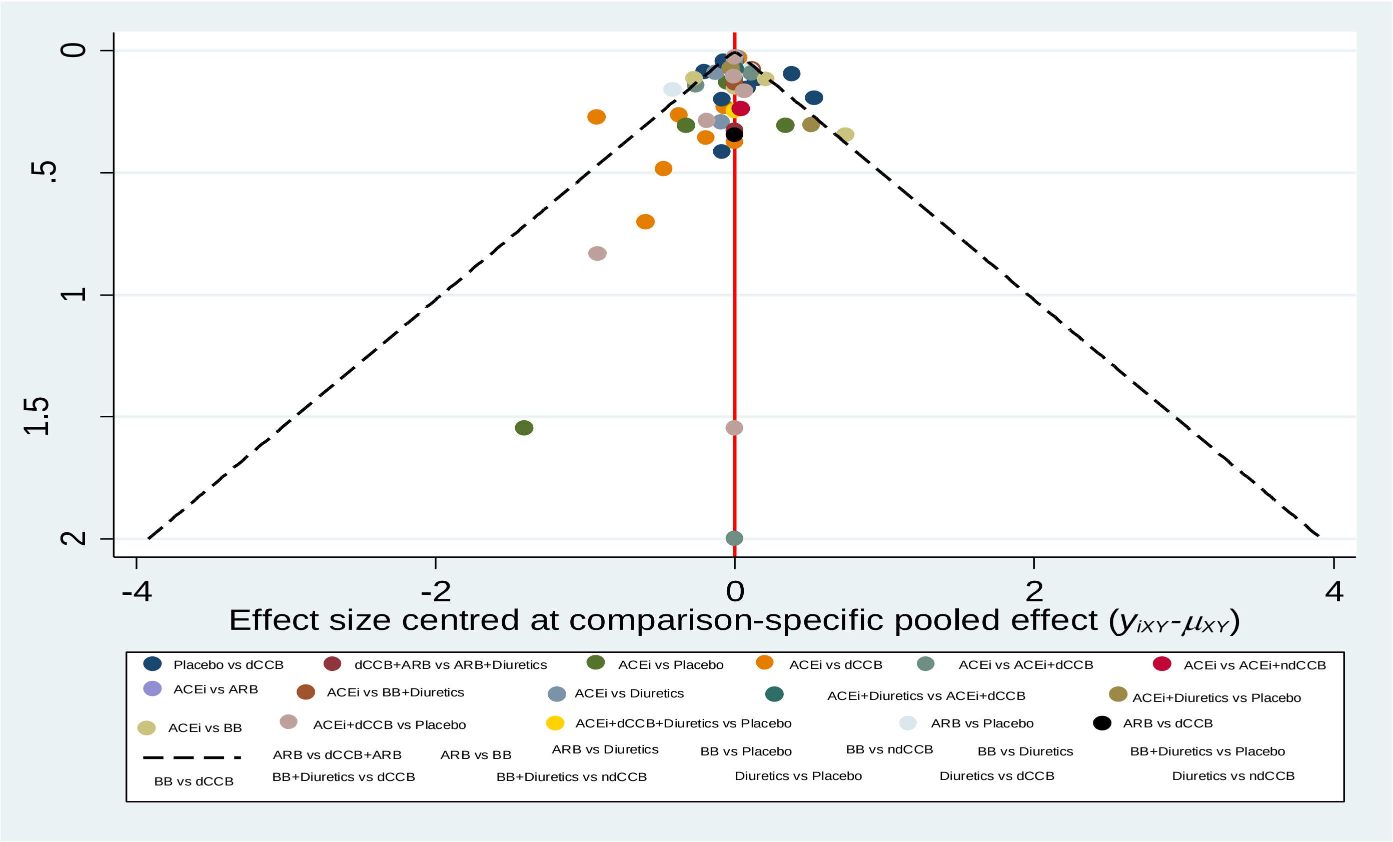

© 2020 Wei J et al. *JAMA Netw Open*. All rights reserved.

eFigure 2. Inconsistency Plots of Studies of Blood Pressure–Lowering Drugs and Effects of Prevention on Cardiovascular Death, Myocardial Infarction, Stroke, Revascularization, and Overall Cardiovascular Events

A. Cardiovascular death

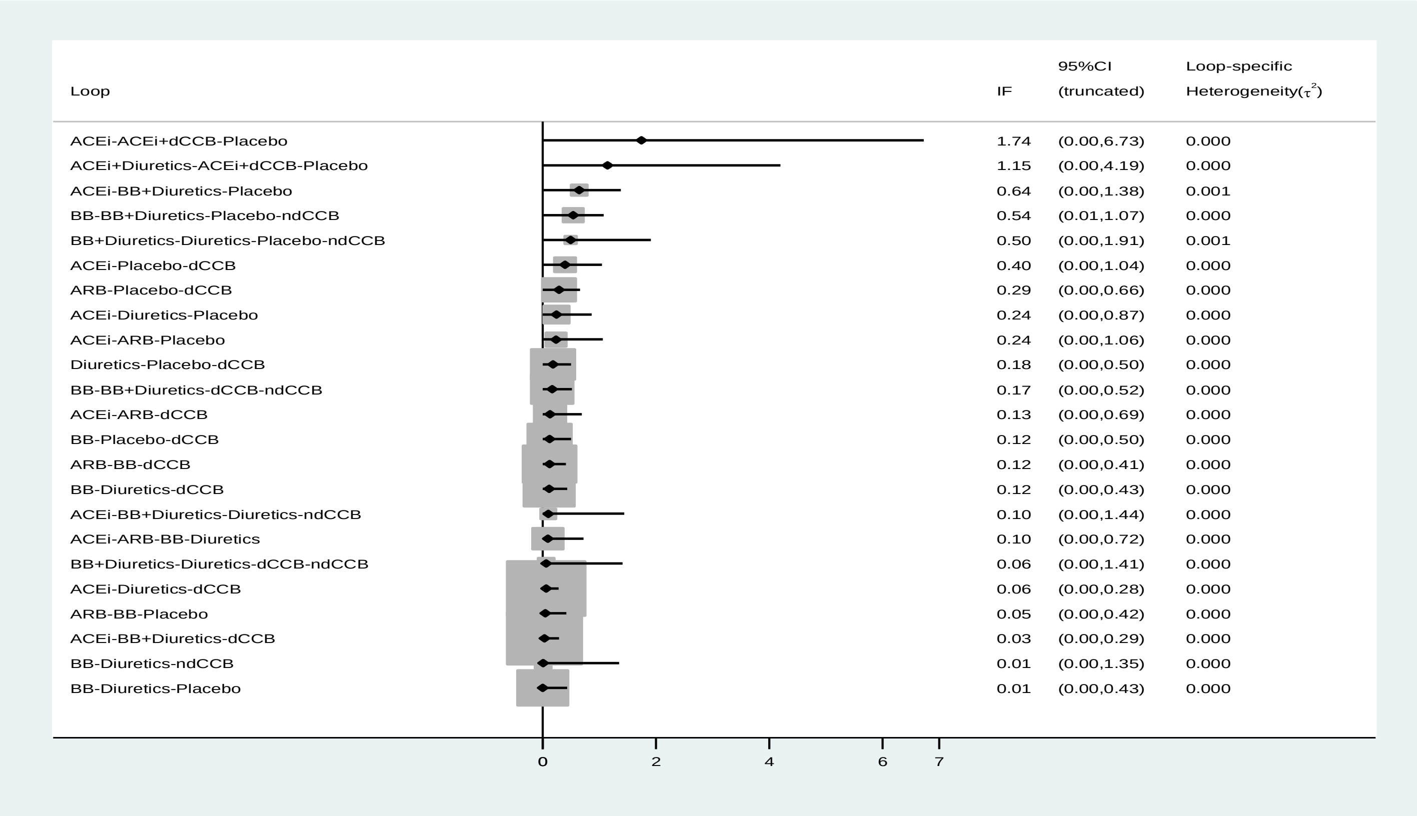

© 2020 Wei J et al. *JAMA Netw Open*. All rights reserved.

B. Myocardial infarction

Loop	IF	95%CI (truncated)	Loop-specific Heterogeneity(τ^2)
ACEi-BB-dCCB	0.47	(0.00,1.14)	0.032
BB+Diuretics-Diuretics-Placebo	0.41	(0.00,1.14)	0.000
ARB-Placebo-dCCB	0.40	(0.00,1.63)	0.149
BB-Diuretics-dCCB	0.36	(0.04,0.68)	0.000
BB-Diuretics-Placebo	0.34	(0.00,0.91)	0.000
ACEi-ARB-dCCB	0.34	(0.00,1.13)	0.034
ACEi-BB-BB+Diuretics-ndCCB	0.34	(0.00,0.85)	0.000
BB-BB+Diuretics-Placebo-ndCCB	0.32	(0.00,1.09)	0.000
ACEi-Diuretics-Placebo	0.31	(0.00,0.80)	0.000
BB-Diuretics-ndCCB	0.28	(0.00,1.57)	0.000
ACEi-Placebo-dCCB	0.28	(0.00,1.09)	0.034
ACEi-ARB-BB	0.25	(0.00,0.91)	0.000
ACEi-Diuretics-dCCB	0.25	(0.00,0.49)	0.000
BB+Diuretics-Placebo-dCCB	0.22	(0.00,1.02)	0.000
BB-Placebo-dCCB	0.21	(0.00,0.82)	0.000
ARB-BB-Placebo	0.20	(0.00,0.72)	0.000
Diuretics-Placebo-dCCB	0.20	(0.00,0.81)	0.000
BB-BB+Diuretics-dCCB-ndCCB	0.11	(0.00,0.52)	0.000
ACEi-BB+Diuretics-dCCB	0.06	(0.00,0.77)	0.044
ACEi-BB+Diuretics-Diuretics	0.06	(0.00,0.50)	0.013
ACEi-ARB-Placebo	0.05	(0.00,0.75)	0.000
ARB-BB-dCCB	0.04	(0.00,0.31)	0.000
ACEi-BB-Diuretics	0.03	(0.00,0.50)	0.000
BB+Diuretics-Diuretics-ndCCB	0.03	(0.00,1.31)	0.000
ACEi-BB+Diuretics-Placebo	0.02	(0.00,0.72)	0.000
BB+Diuretics-Diuretics-dCCB	0.01	(0.00,0.32)	0.000

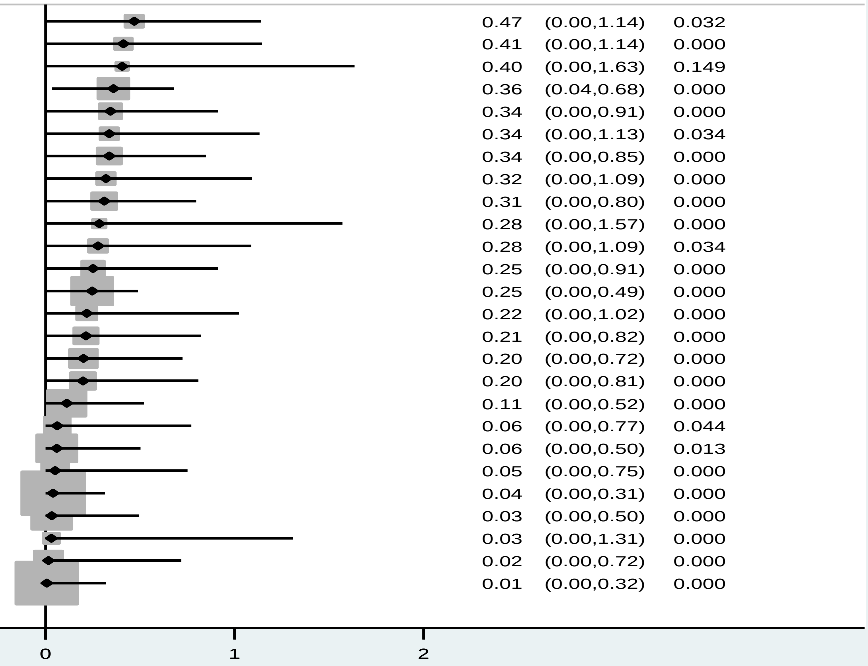

*** Loop(s) [ACEi-BB-Placebo] are formed only by multi-arm trial(s) - Consistent by definition

© 2020 Wei J et al. *JAMA Netw Open*. All rights reserved.

C. Stroke

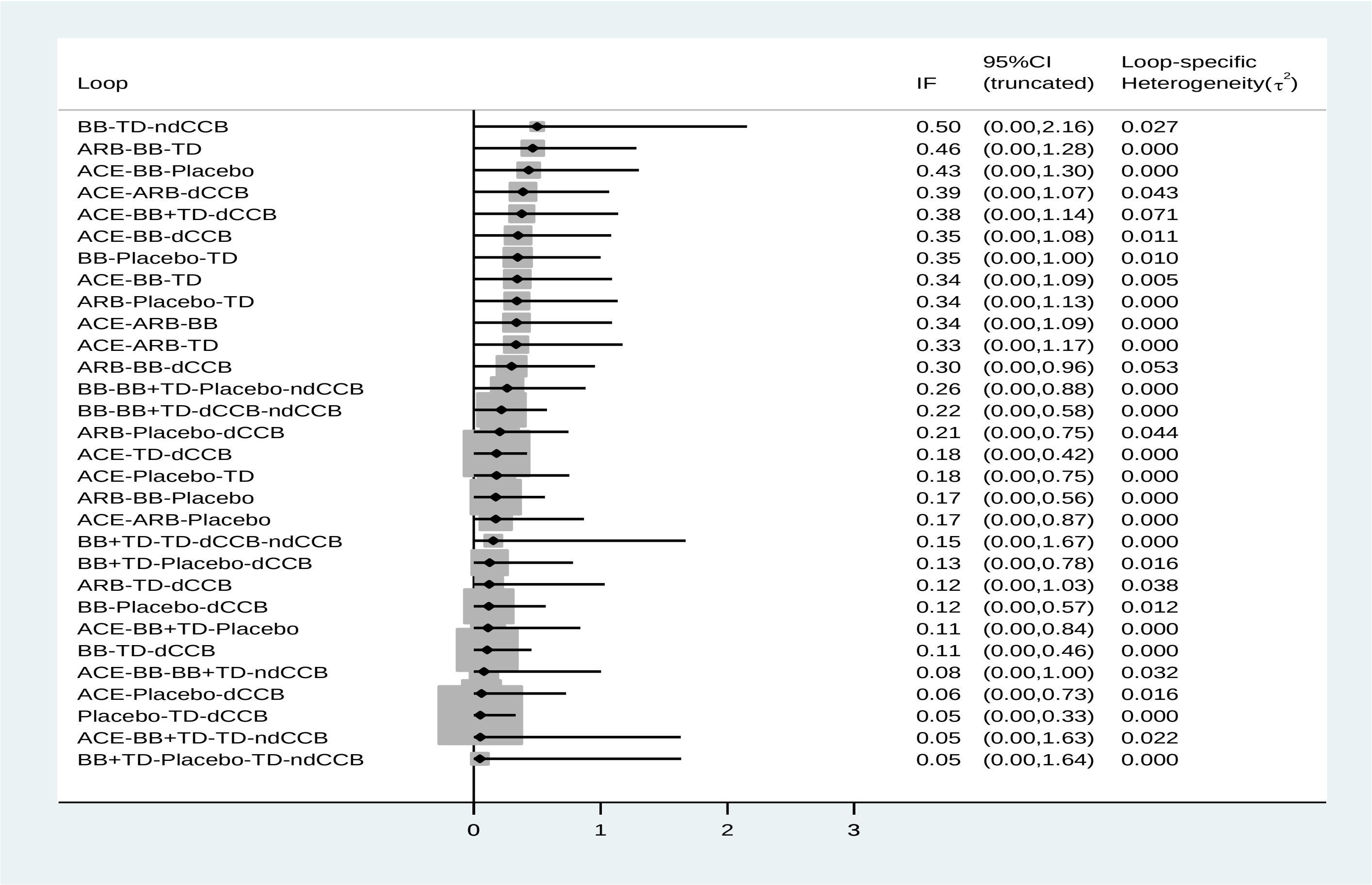

D. Revascularization

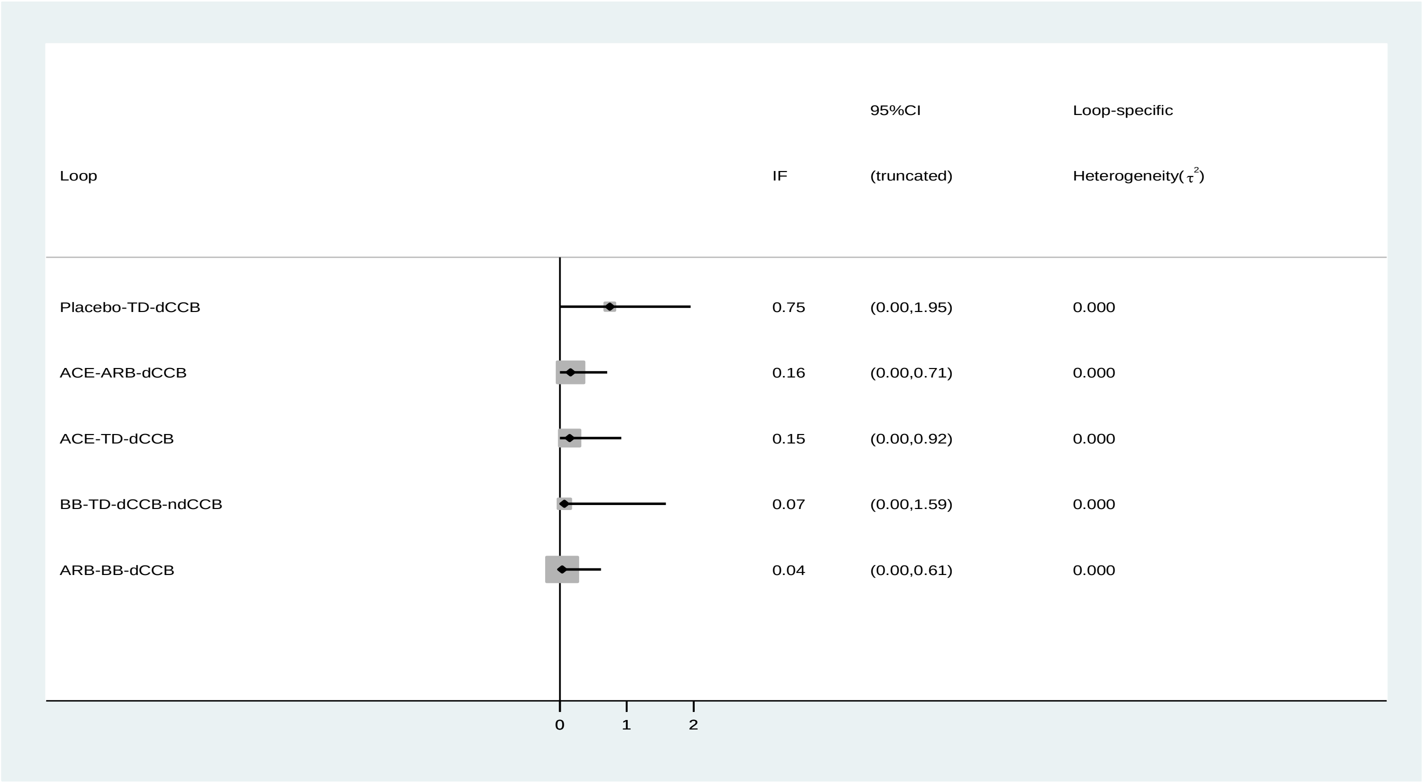

© 2020 Wei J et al. *JAMA Netw Open*. All rights reserved.

E. Overall cardiovascular events

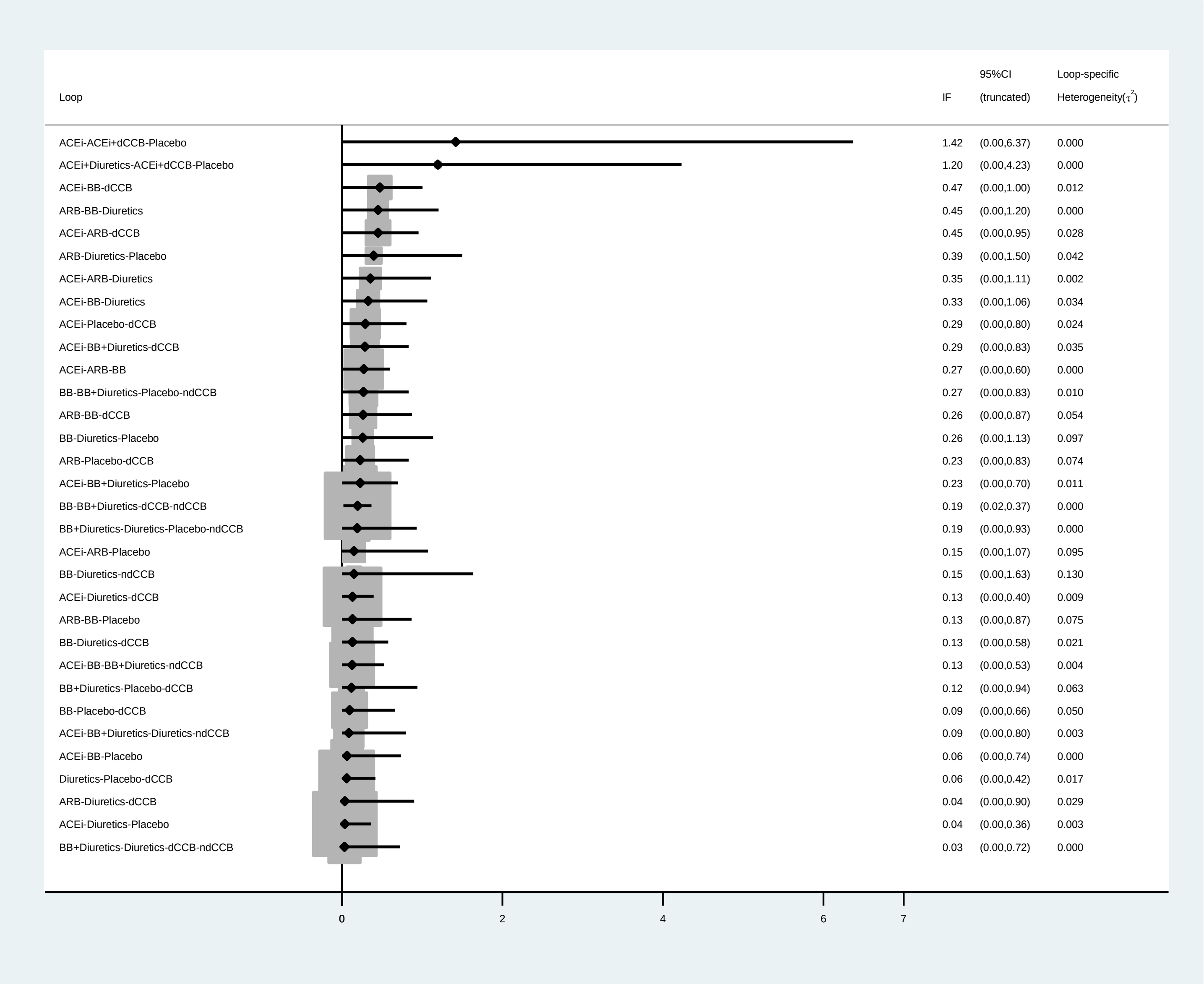

© 2020 Wei J et al. *JAMA Netw Open*. All rights reserved.

eReferences

1. Ruggenenti P, Lauria G, Iliev IP, et al. Effects of manidipine and delapril in hypertensive patients with type 2 diabetes mellitus: the delapril and manidipine for nephroprotection in diabetes (DEMAND) randomized clinical trial. *Hypertension.* 2011;58(5):776-783.

2. Liu L, Zhang Y, Liu G, Li W, Zhang X, Zanchetti A. The Felodipine Event Reduction (FEVER) Study: a randomized long-term placebo-controlled trial in Chinese hypertensive patients. *Journal of hypertension.* 2005;23(12):2157-2172.

3. Medical Research Council trial of treatment of hypertension in older adults: principal results. MRC Working Party. *BMJ (Clinical research ed).* 1992;304(6824):405-412.

4. Prevention of stroke by antihypertensive drug treatment in older persons with isolated systolic hypertension. Final results of the Systolic Hypertension in the Elderly Program (SHEP). SHEP Cooperative Research Group. *JAMA : the journal of the American Medical Association.* 1991;265(24):3255-3264.

5. Dahlof B, Lindholm LH, Hansson L, Schersten B, Ekbom T, Wester PO. Morbidity and mortality in the Swedish Trial in Old Patients with Hypertension (STOP-Hypertension). *Lancet.* 1991;338(8778):1281-1285.

6. Liu L, Wang JG, Gong L, Liu G, Staessen JA. Comparison of active treatment and placebo in older Chinese patients with isolated systolic hypertension. Systolic Hypertension in China (Syst-China) Collaborative Group. *Journal of hypertension.* 1998;16(12 Pt 1):1823-1829.

7. Staessen JA, Fagard R, Thijs L, et al. Randomised double-blind comparison of placebo and active treatment for older patients with isolated systolic hypertension. The Systolic Hypertension in Europe (Syst-Eur) Trial Investigators. *Lancet.* 1997;350(9080):757-764.

© 2020 Wei J et al. *JAMA Netw Open*. All rights reserved.

8. Sun M, Zhou H, Jia Z. [Prevention and treatment of stroke after hypertension for ten years in Hunan Province]. *Zhonghua nei ke za zhi.* 1997;36(5):312-314.

9. Sun ZQ, Zheng LQ, Zhang DY, et al. [Analysis of therapeutic effects of rural patients with hypertension by combination administration of low dosage of hydrochlorothiazide and nitrendipine]. *Zhonghua Xin Xue Guan Bing Za Zhi.* 2010;38(2):135-138.

10. Bulpitt CJ, Beckett NS, Cooke J, et al. Results of the pilot study for the Hypertension in the Very Elderly Trial. *Journal of hypertension.* 2003;21(12):2409-2417.

11. Efficacy of atenolol and captopril in reducing risk of macrovascular and microvascular complications in type 2 diabetes: UKPDS 39. UK Prospective Diabetes Study Group. *BMJ (Clinical research ed).* 1998;317(7160):713-720.

12. Tight blood pressure control and risk of macrovascular and microvascular complications in type 2 diabetes: UKPDS 38. UK Prospective Diabetes Study Group. *BMJ (Clinical research ed).* 1998;317(7160):703-713.

13. Estacio RO, Jeffers BW, Hiatt WR, Biggerstaff SL, Gifford N, Schrier RW. The effect of nisoldipine as compared with enalapril on cardiovascular outcomes in patients with non-insulin-dependent diabetes and hypertension. *The New England journal of medicine.* 1998;338(10):645-652.

14. Kjeldsen SE, Jamerson KA, Bakris GL, et al. Predictors of blood pressure response to intensified and fixed combination treatment of hypertension: the ACCOMPLISH study. *Blood pressure.* 2008;17(1):7-17.

15. Major outcomes in high-risk hypertensive patients randomized to angiotensin-converting enzyme inhibitor or calcium channel blocker vs diuretic: The Antihypertensive and Lipid-Lowering Treatment to Prevent Heart Attack Trial (ALLHAT). *JAMA : the journal of the American Medical Association.* 2002;288(23):2981-2997.

© 2020 Wei J et al. *JAMA Netw Open*. All rights reserved.

16. Dahlof B, Sever PS, Poulter NR, et al. Prevention of cardiovascular events with an antihypertensive regimen of amlodipine adding perindopril as required versus atenolol adding bendroflumethiazide as required, in the Anglo-Scandinavian Cardiac Outcomes Trial-Blood Pressure Lowering Arm (ASCOT-BPLA): a multicentre randomised controlled trial. *Lancet.* 2005;366(9489):895-906.

17. Ruggenenti P, Fassi A, Ilieva A, et al. Effects of verapamil added-on trandolapril therapy in hypertensive type 2 diabetes patients with microalbuminuria: the BENEDICT-B randomized trial. *Journal of hypertension.* 2011;29(2):207-216.

18. Ogihara T, Saruta T, Rakugi H, et al. Combinations of olmesartan and a calcium channel blocker or a diuretic in elderly hypertensive patients: a randomized, controlled trial1. *Journal of hypertension.* 2014.

19. Black HR, Elliott WJ, Grandits G, et al. Principal results of the Controlled Onset Verapamil Investigation of Cardiovascular End Points (CONVINCE) trial. *JAMA : the journal of the American Medical Association.* 2003;289(16):2073-2082.

20. Zanchetti A, Bond MG, Hennig M, et al. Calcium antagonist lacidipine slows down progression of asymptomatic carotid atherosclerosis: principal results of the European Lacidipine Study on Atherosclerosis (ELSA), a randomized, double-blind, long-term trial. *Circulation.* 2002;106(19):2422-2427.

21. Tatti P, Pahor M, Byington RP, et al. Outcome results of the Fosinopril Versus Amlodipine Cardiovascular Events Randomized Trial (FACET) in patients with hypertension and NIDDM. *Diabetes care.* 1998;21(4):597-603.

22. A 12-month comparison of ACE inhibitor and CA antagonist therapy in mild to moderate essential hypertension--The GLANT Study. Study Group on Long-term Antihypertensive Therapy. *Hypertension research : official journal of the Japanese Society of Hypertension.* 1995;18(3):235-244.

© 2020 Wei J et al. *JAMA Netw Open*. All rights reserved.

23. Brown MJ, Palmer CR, Castaigne A, et al. Morbidity and mortality in patients randomised to double-blind treatment with a long-acting calcium-channel blocker or diuretic in the International Nifedipine GITS study: Intervention as a Goal in Hypertension Treatment (INSIGHT). *Lancet.* 2000;356(9227):366-372.

24. Pepine CJ, Handberg EM, Cooper-DeHoff RM, et al. A calcium antagonist vs a non-calcium antagonist hypertension treatment strategy for patients with coronary artery disease. The International Verapamil-Trandolapril Study (INVEST): a randomized controlled trial. *JAMA : the journal of the American Medical Association.* 2003;290(21):2805-2816.

25. Baba S. Nifedipine and enalapril equally reduce the progression of nephropathy in hypertensive type 2 diabetics. *Diabetes research and clinical practice.* 2001;54(3):191-201.

26. Yui Y, Sumiyoshi T, Kodama K, et al. Comparison of nifedipine retard with angiotensin converting enzyme inhibitors in Japanese hypertensive patients with coronary artery disease: the Japan Multicenter Investigation for Cardiovascular Diseases-B (JMIC-B) randomized trial. *Hypertension research : official journal of the Japanese Society of Hypertension.* 2004;27(3):181-191.

27. Dahlof B, Devereux RB, Kjeldsen SE, et al. Cardiovascular morbidity and mortality in the Losartan Intervention For Endpoint reduction in hypertension study (LIFE): a randomised trial against atenolol. *Lancet.* 2002;359(9311):995-1003.

28. Wikstrand J, Warnold I, Tuomilehto J, et al. Metoprolol versus thiazide diuretics in hypertension. Morbidity results from the MAPHY Study. *Hypertension.* 1991;17(4):579-588.

29. Borhani NO, Mercuri M, Borhani PA, et al. Final outcome results of the Multicenter Isradipine Diuretic Atherosclerosis Study (MIDAS). A randomized controlled trial. *JAMA : the journal of the American Medical Association.* 1996;276(10):785-791.

© 2020 Wei J et al. *JAMA Netw Open*. All rights reserved.

30. Schrader J, Luders S, Kulschewski A, et al. Morbidity and Mortality After Stroke, Eprosartan Compared with Nitrendipine for Secondary Prevention: principal results of a prospective randomized controlled study (MOSES). *Stroke; a journal of cerebral circulation.* 2005;36(6):1218-1226.

31. Muramatsu T, Matsushita K, Yamashita K, et al. Comparison between valsartan and amlodipine regarding cardiovascular morbidity and mortality in hypertensive patients with glucose intolerance: NAGOYA HEART Study. *Hypertension.* 2012;59(3):580-586.

32. Randomized double-blind comparison of a calcium antagonist and a diuretic in elderly hypertensives. National Intervention Cooperative Study in Elderly Hypertensives Study Group. *Hypertension.* 1999;34(5):1129-1133.

33. Hansson L, Hedner T, Lund-Johansen P, et al. Randomised trial of effects of calcium antagonists compared with diuretics and beta-blockers on cardiovascular morbidity and mortality in hypertension: the Nordic Diltiazem (NORDIL) study. *Lancet.* 2000;356(9227):359-365.

34. Ogawa H, Kim-Mitsuyama S, Matsui K, Jinnouchi T, Jinnouchi H, Arakawa K. Angiotensin II receptor blocker-based therapy in Japanese elderly, high-risk, hypertensive patients. *Am J Med.* 2012;125(10):981-990.

35. Ogihara T. Practitioner's Trial on the Efficacy of Antihypertensive Treatment in the Elderly Hypertension (The PATE-Hypertension Study) in Japan. *American journal of hypertension.* 2000;13(5 Pt 1):461-467.

36. Malacco E, Mancia G, Rappelli A, Menotti A, Zuccaro MS, Coppini A. Treatment of isolated systolic hypertension: the SHELL study results. *Blood pressure.* 2003;12(3):160-167.

37. Hansson L, Lindholm LH, Ekbom T, et al. Randomised trial of old and new antihypertensive drugs in elderly patients: cardiovascular mortality and morbidity the Swedish Trial in Old Patients with Hypertension-2 study. *Lancet.* 1999;354(9192):1751-1756.

© 2020 Wei J et al. *JAMA Netw Open*. All rights reserved.

38. Julius S, Kjeldsen SE, Weber M, et al. Outcomes in hypertensive patients at high cardiovascular risk treated with regimens based on valsartan or amlodipine: the VALUE randomised trial. *Lancet.* 2004;363(9426):2022-2031.

39. Narumi H, Takano H, Shindo S, et al. Effects of valsartan and amlodipine on cardiorenal protection in Japanese hypertensive patients: the Valsartan Amlodipine Randomized Trial. *Hypertension research : official journal of the Japanese Society of Hypertension.* 2011;34(1):62-69.

40. Rosei EA, Dal Palu C, Leonetti G, Magnani B, Pessina A, Zanchetti A. Clinical results of the Verapamil inHypertension and Atherosclerosis Study. VHAS Investigators. *Journal of hypertension.* 1997;15(11):1337-1344.

41. Wing LM, Reid CM, Ryan P, et al. A comparison of outcomes with angiotensin-converting--enzyme inhibitors and diuretics for hypertension in the elderly. *The New England journal of medicine.* 2003;348(7):583-592.

42. Matsuzaki M, Ogihara T, Umemoto S, et al. Prevention of cardiovascular events with calcium channel blocker-based combination therapies in patients with hypertension: a randomized controlled trial. *Journal of hypertension.* 2011;29(8):1649-1659.

43. Suzuki H, Kanno Y. Effects of candesartan on cardiovascular outcomes in Japanese hypertensive patients. *Hypertension research : official journal of the Japanese Society of Hypertension.* 2005;28(4):307-314.

44. Beckett N, Peters R, Leonetti G, et al. Subgroup and per-protocol analyses from the Hypertension in the Very Elderly Trial. *Journal of hypertension.* 2014;32(7):1478-1487; discussion 1487.

45. Hansson L, Lindholm LH, Niskanen L, et al. Effect of angiotensin-converting-enzyme inhibition compared with conventional therapy on cardiovascular morbidity and mortality in hypertension: the Captopril Prevention Project (CAPPP) randomised trial. *Lancet.* 1999;353(9153):611-616.

© 2020 Wei J et al. *JAMA Netw Open*. All rights reserved.

46. Lithell H, Hansson L, Skoog I, et al. The Study on Cognition and Prognosis in the Elderly (SCOPE): principal results of a randomized double-blind intervention trial. *Journal of hypertension.* 2003;21(5):875-886.

47. Kasanuki H, Hagiwara N, Hosoda S, et al. Angiotensin II receptor blocker-based vs. non-angiotensin II receptor blocker-based therapy in patients with angiographically documented coronary artery disease and hypertension: the Heart Institute of Japan Candesartan Randomized Trial for Evaluation in Coronary Artery Disease (HIJ-CREATE). *European heart journal.* 2009;30(10):1203-1212.

© 2020 Wei J et al. *JAMA Netw Open*. All rights reserved.
